# Phylogenetic Species Identification in *Rattus* Highlights Rapid Radiation and Morphological Similarity of New Guinean Species

**DOI:** 10.1371/journal.pone.0098002

**Published:** 2014-05-27

**Authors:** Judith H. Robins, Vernon Tintinger, Ken P. Aplin, Melanie Hingston, Elizabeth Matisoo-Smith, David Penny, Shane D. Lavery

**Affiliations:** 1 School of Biological Sciences and Department of Anthropology, The University of Auckland, Auckland, New Zealand; 2 Department of Anthropology, The University of Auckland, Auckland, New Zealand; 3 Division of Mammals, National Museum of Natural History, Smithsonian Institution, Washington, DC, United States of America; 4 School of Biological Sciences, The University of Auckland, Auckland, New Zealand; 5 Department of Anatomy, University of Otago, Dunedin, New Zealand; 6 Institute of Fundamental Sciences, Massey University, Palmerston North, New Zealand; 7 School of Biological Sciences and Institute of Marine Science, The University of Auckland, Auckland, New Zealand; Swansea University, United Kingdom

## Abstract

The genus *Rattus* is highly speciose, the taxonomy is complex, and individuals are often difficult to identify to the species level. Previous studies have demonstrated the usefulness of phylogenetic approaches to identification in *Rattus* but some species, especially among the endemics of the New Guinean region, showed poor resolution. Possible reasons for this are simple misidentification, incomplete gene lineage sorting, hybridization, and phylogenetically distinct lineages that are unrecognised taxonomically. To assess these explanations we analysed 217 samples, representing nominally 25 *Rattus* species, collected in New Guinea, Asia, Australia and the Pacific. To reduce misidentification problems we sequenced museum specimens from earlier morphological studies and recently collected tissues from samples with associated voucher specimens. We also reassessed vouchers from previously sequenced specimens. We inferred combined and separate phylogenies from two mitochondrial DNA regions comprising 550 base pair D-loop sequences and both long (655 base pair) and short (150 base pair) cytochrome oxidase I sequences. Our phylogenetic species identification for 17 species was consistent with morphological designations and current taxonomy thus reinforcing the usefulness of this approach. We reduced misidentifications and consequently the number of polyphyletic species in our phylogenies but the New Guinean *Rattus* clades still exhibited considerable complexity. Only three of our eight New Guinean species were monophyletic. We found good evidence for either incomplete mitochondrial lineage sorting or hybridization between species within two pairs, *R. leucopus*/*R*. cf. *verecundus* and *R. steini/R*. *praetor*. Additionally, our results showed that *R. praetor*, *R. niobe* and *R*. *verecundus* each likely encompass more than one species. Our study clearly points to the need for a revised taxonomy of the rats of New Guinea, based on broader sampling and informed by both morphology and phylogenetics. The remaining taxonomic complexity highlights the recent and rapid radiation of *Rattus* in the Australo-Papuan region.

## Introduction

With more than 60 currently recognised species, the genus *Rattus* features large in the native rodent fauna of mainland Asia, Island South East Asia, Australia and Melanesia [1]. The genus probably originated on mainland Asia [2], [3] but there has been a successful invasion of New Guinea and Australia which harbour about 20 endemic species [1], [4], [5]. Although the majority of *Rattus* species are restricted to natural habitats within their native ranges, many seem to thrive in disturbed habitats, and a significant number have become agricultural pests, especially in Asia [6]. Two species, *R. rattus* and *R. norvegicus*, became commensal and achieved an almost world-wide distribution largely via European sailing ships [7], while a third commensal, *R. exulans*, was distributed throughout the Pacific via the canoes of prehistoric Pacific colonists and traders [8]. The two most widespread commensal species (*R. rattus* and *R. norvegicus*) are known to play a key role in important zoonotic disease cycles [9], while *R. rattus* and *R. exulans* are ecologically invasive and have had devastating effects on native biota, particularly on islands [10], [11], [12].

Accurate identification of *Rattus* to the species level is important in numerous contexts including autecological and community ecology studies, the design and implementation of both conservation and pest management programs, and the investigation of zoonotic disease cycles. Even though species of *Rattus* are encountered more often than any other group of small mammals in the Asia-Pacific region, they are notoriously difficult to identify in the field, even in reliably distinguishing introduced from native species [6]. This difficulty stems from a combination of intrinsic morphological conservatism, substantial changes in pelage colour and texture through life, and an unusual level of plasticity in both phenotypic and reproductive characters in species that live under multiple bioclimatic regimes [6].

Phylogenetic (i.e. gene-tree based) methods offer considerable promise for both species identification [13], [14], [15], [16] and species delimitation [17], [18], [19] in speciose, but morphologically conservative, taxa. While single genes may be sufficient for species identification, multigene approaches are necessary for species delimitation [20]. Several recent studies have employed phylogenetic methods to identify and delimit species in rodents including *Rattus* [9], [21], [22], [23], [24]. Robins et al. [24] used D-loop, cytochrome *b* (cyt *b*) and cytochrome oxidase I (COI) sequences in a study that focussed on identification of multiple Asian and Australo-Papuan *Rattus* species, while Pagès et al. [23] used sequences of cyt *b*, COI and the nuclear interphotoreceptor retinoid-binding protein gene (IRBP) in a wider study of the Asian members of the Tribe Rattini (a grouping of *Rattus*-like genera below the level of family [25]). Cyt *b* sequences were used to identify invasive *Rattus* species in South Africa [21] and to assess the taxonomic status of Asian rats with particular emphasis on *R*. *rattus* [9]. In another relevant study, Rowe et al. [26] analysed phylogenetic relationships among Australian and New Guinean *Rattus* using sequences of D-loop and nine nuclear genes from representatives of eight species.

In all of these studies of *Rattus* and its close allies there are instances of mismatch between specimens previously identified based on morphology and their placement on gene trees. These mismatches preclude a simple phylogenetic resolution of all *Rattus* species and may arise for one or more of the following reasons:

1. simple cases of misidentification based on inadequate morphological assessment making phylogenetic clades appear polyphyletic when they are not;

2. curatorial confusion that has arisen due to the complex taxonomic history of many groups and the occurrence of numerous synonyms (e.g. over 80 for *R. rattus* [1]);

3. instances where morphological identification is correct but gene tree topology is confounded by incomplete lineage sorting and/or hybridisation among species;

4. the presence of cryptic species (i.e. phylogenetically distinct lineages that have not been recognised as taxonomically distinct);

5. the occurrence of pseudogenes or numts (i.e. nuclear paralogues of mitochondrial gene sequences).

Robins et al. [24] found numerous instances of mismatch between nominal identity and mitochondrial affinity within *Rattus*, where ‘nominal’ is defined as the sample identification given by the collector or museum. The mismatch frequency was especially high among the New Guinean native *Rattus* which represent a particularly rapid speciation probably within one million years [26], [27]. Robins et al. [24] suspected that while misidentification played an important part in these mismatch problems, it was unlikely to be the full story.

In this paper we explore the extent to which the native *Rattus* species of the New Guinean region are monophyletic on phylogenetic trees estimated using mitochondrial genes, and consequently the usefulness of these genes in identifying members of these species. When species are not monophyletic, we explore possible causes. The question of what should be the species boundaries is beyond the scope of this paper and requires the use of nuclear loci in addition to the mitochondrial loci and morphological characterisations considered here.

In the last major morphology-based revision of this group, Taylor et al. [5] recognised 11 native *Rattus* species in New Guinea (including 23 subspecies) and five introduced species. The native species were placed into three groups: 1. *R. niobe* (two subspecies), *R. richardsoni* and *R. verecundus* (four subspecies); 2. *R. praetor* (two subspecies), *R. mordax* (two subspecies), *R. leucopus* (three subspecies), *R. steini* (four subspecies), *R. giluwensis, R. novaeguineae* and *R. jobiensis*; and 3. *R. sordidus* (two subspecies). The members of the first group were later removed from *Rattus* and placed in the genus *Stenomys* because of their unusual morphology and adaptations [28]. Flannery [29] and others followed this usage but later, in the light of molecular systematics, Musser and Carleton [1] transferred *Stenomys* back into *Rattus*. They included all New Guinean species of *Rattus* as members of an ‘*R. leucopus* species group’, except for *R. sordidus* which they placed in an ‘*R fuscipe*s species group’. In addition they considered *R. omichlodes* to be a separate species from *R. richardsoni* and *R. niobe* to be a complex comprising three or four species (*R. niobe, R. arrogans, R. pococki* and provisionally *R. arfakiensis*). In this arrangement, *R. niobe* (*sensu stricto*) is restricted to the mountains of Papua New Guinea (the eastern half of the island of New Guinea) whereas the other three species are distributed in the western half of the island including the Indonesian Province of Papua (previously known as Irian Jaya). Our study builds on previous molecular studies of Australo-Papuan *Rattus* of Robins et al., [24], [27] and Rowe et al. [26].

In order to minimise the problem of simple misidentification in the field or museum, we focussed our effort on New Guinean *Rattus* specimens that either were included in the detailed morphological appraisal of Taylor et al. [5] or were available as recently collected tissue samples with associated voucher specimens that could be critically assessed. For the first category of specimens, we obtained samples of bone and skin from specimens critically examined and identified by Mary Taylor and her co-workers [5]. Their analysis was based on a total of 7,580 specimens. The measurements taken were: 20 from each skull; head plus body length; tail length; and hind foot length - although not all measurements were possible for every sample. Pelage colour and texture were also assessed for recent samples that had not been subjected to spirit preservation. For the second category, we obtained liver samples from specimens collected since the advent of routine tissue sampling of New Guinean vertebrates, which commenced in earnest in the early 1980s. To establish a broad framework for the investigation of New Guinean *Rattus*, we also compiled a larger dataset that included many other species of *Rattus* from Asia to Australia, using sequences published by Robins et al. [24], [27], Rowe et al. [26], and others.

The use of samples derived from critically identified specimens improves the resolution of some species and points to probable instances of incomplete mitochondrial lineage sorting and/or hybridisation with mitochondrial introgression. Further, our study indicates the likely presence of several currently unrecognised species and thus emphasizes the need for a combined molecular-morphological taxonomic revision of New Guinea *Rattus*. Analysis of the larger dataset also highlights some taxonomic misidentifications within recently published molecular work on Asian *Rattus*.

## Methods

**Figure 1:**
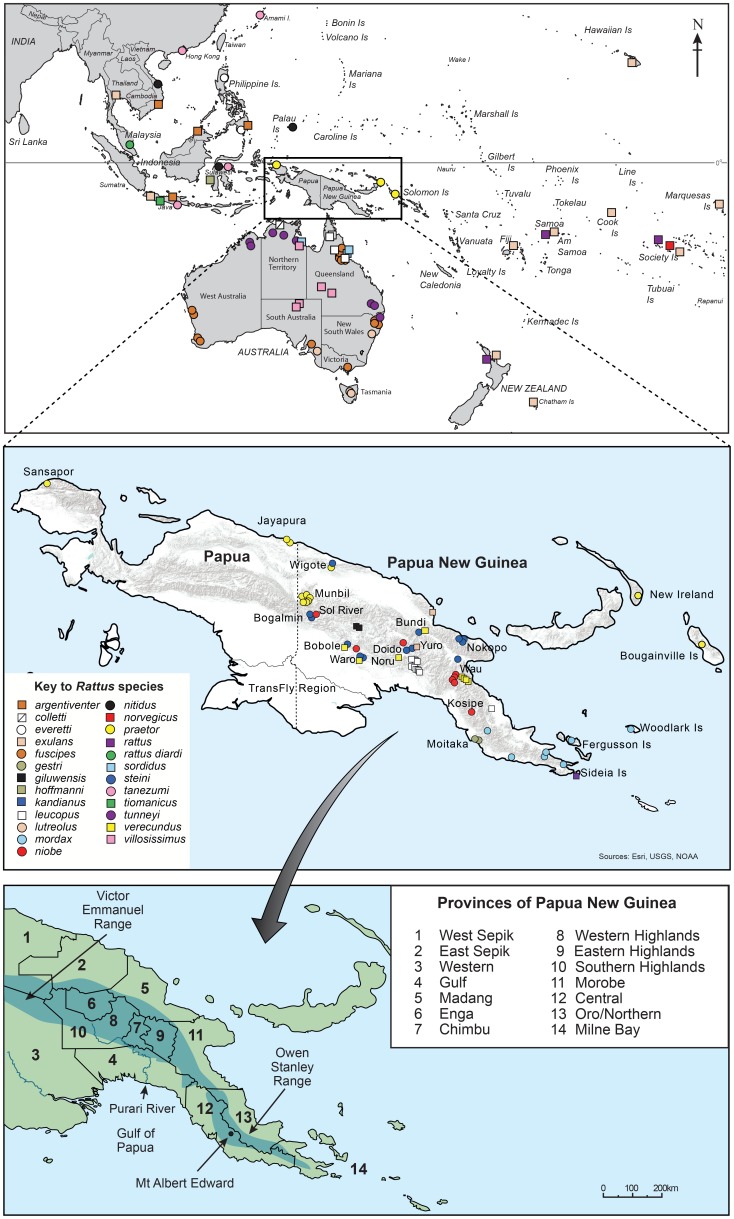
Sample location map showing South East Asia, Australia, New Guinea and the western Pacific region. The middle pane is a more detailed view of New Guinea (comprising Papua, a province of Indonesia, and Papua New Guinea) and the bottom pane is a map of Papua New Guinea showing some major features, including the provinces, mentioned in the text. Note that given the scales involved the sample positions are approximate.

We analysed data from a total of 217 samples representing nominally 25 *Rattus* species and three species of other genera of Rattini (*Leopoldamys sabanus*, *Niviventer fulvescens* and *Sundamys muelleri*) which were used as outgroups. See Fig. 1 for sample locations. We acquired small fragments of turbinal bone/nasal cartilage and/or skin with attached fur from historical New Guinean *Rattus* specimens held in the American Museum of Natural History, New York (AMNH: 7 samples), the United States National Museum, Smithsonian Institution (USNM: 29 samples) and the Australian National Wildlife Collection, CSIRO, Canberra (ANWC: 4 samples). We obtained ethanol preserved liver samples from New Guinean *Rattus* from the Australian Biological Tissue Collection, South Australian Museum (ABTC: 15 samples). Sequences from a total of 33 specimens of New Guinean *Rattus* and 41 Australian *Rattus* were included from the publications of Robins et al. [24], [27] and Rowe et al. [26]. See Table S1 for details of the samples new to this study and Table S2 for those from previous studies. To avoid problems associated with missing data we excluded sequences from Rowe at al. [26] that gave incomplete coverage of the D-loop region that we had sequenced. For the majority of the New Guinean samples, a morphological voucher was available in the collection of the Australian Museum, Sydney (AM). These were examined first-hand by Aplin and Robins in the context of published accounts [1], [5], [29] and the larger specimen holdings of the Australian Museum; this process resulted in ten changes to species identifications (see Table 1). Many of the specimens were juvenile or sexually immature animals and the original identifications may have failed to take this factor into account. Additional sequences from GenBank from the studies of Balakirev and Rozhnov [30], Nilsson et al. [31], and Pagès et al. [23] were included in our analyses (see Table S2).

**Table 1:** Sample identification history.

Corrected ID	Code in analyses	AMSV #	SAM #	Location where captured	Previous ID
*R. exulans*	*	AMSM18585	ABTC49242	Papua New Guinea, Madang Province, Bundi	*R. steini*
*R. exulans*	*	AMSM14580	ABTC44179	Papua New Guinea, West Sepik Province, Wigote	*R. verecundus*
*R. sordidus gestri*	GePN009	AMSM16320	ABTC44857	Papua New Guinea, National Capital District, Moitaka	*R. rattus*
*R. sordidus gestri*	GePN079	AMSM16319	ABTC44858	Papua New Guinea, National Capital District, Moitaka	*R. rattus*
*R. steini*	StPN054	SAMAM15123	ABTC45756	Papua New Guinea, Southern Highlands Province, Bobole	*R. niobe*
*R. steini*	StPN072	AMSM20028	ABTC49306	Papua New Guinea, Madang Province, Bundi	*R. niobe*
*R. steini*	StPN046	AMSM19055	ABTC48962	Papua New Guinea, Morobe Province, Nokopo	*R. mordax, R. novaeguineae*
*R. steini*	StPN051	AMSM16318	ABTC46853	Papua New Guinea, Southern Highlands Province, Waro	*R. novaeguineae*
*R. steini*	StPN055	AMSM14647	ABTC43216	Papua New Guinea, Chimbu Province, Yuro	*R. ruber, R. novaeguineae*
*R. steini*	StPN095	AMSM19056	ABTC48963	Papua New Guinea, Morobe Province, Nokopo	*R steini, R. novaeguineae*

ID is the species identification. AMSV # is the voucher number used by the Australian Museum Sydney. SAM # is the tissue accession number used by the South Australian Museum.

*indicates that the vouchers were examined but the samples were not included in the current analyses because their sequences are the same haplotype as *R. exulans* ExPN025 which was included.

### DNA extraction amplification and sequencing

Different methods were used to process the three tissue types; modern tissues, ancient bone, or snips of dried skin from historic museum voucher specimens.

DNA from modern tissue was extracted from muscle, liver or tail samples preserved in 70% ethanol using either standard phenol chloroform methods [32] or the High Pure PCR Template Preparation Kit from Roche. Ancient and historic samples were processed in a dedicated ancient DNA facility in the Department of Anthropology at the University of Auckland (see later). We developed a simplified guanidinium thiocyanate (GuSCN) and silica extraction procedure for the bones and we modified this method further for the extraction of DNA from the dried skin fragments.

### Ancient DNA lab extractions

Ancient bone and historic skin samples were extracted and PCRs were set up in our ancient DNA laboratory. Standard precautions were taken to protect against contamination [33]. The laboratory is physically separate from all post-PCR activity and the workflow is unidirectional beginning in the ancient lab where no amplified products have ever been. Samples were processed in small batches of no more than five at a time, with negative control extractions always included. The PCR controls included attempts to amplify the negative extractions as well as standard template free negatives.

Ancient bone samples were processed using a silica/GuSCN protocol modified from Rohland and Hofreiter [34], Matisoo-Smith et al. [35] and Höss and Pääbo [36]. 1 mL of a digestion buffer (0.5 M EDTA pH 8.0, 1.6% Triton X-100) and 20 µL proteinase K (20 mg/mL) was added to 50 mg ground bone in a sterile 2 mL tube and the sample was rotated overnight at 37°. If undigested bone was still present in the morning, rotation was continued for 1 to 3 hours but the temperature was increased to 56°. After digestion was complete, the tube was centrifuged at 6,700×g for 1 min and 500 µL of the supernatant was transferred for extraction to a newly prepared tube containing 1 mL of DNA binding solution (5 M GuSCN and 25 mM NaCl) and 100 µL of a silica suspension prepared as in [34]. The remaining digest was stored as a backup at −20°. The extraction tube was incubated under rotation at 37° for 3 hours. The tube was centrifuged at 6,700×g for 1 min and the supernatant discarded. The silica pellet was resuspended and washed in 1 mL of the DNA binding solution, followed by two washes in 70% ethanol. After each wash the tube was centrifuged and the supernatant discarded. After the last wash the pellet was dried for 10 minutes at 37° then resuspended in 150 µL of TE buffer (10 mM Tris HCl pH 8.0 and 1 mM EDTA Na_2_ pH 8). The final centrifugation step was at 11,300×g for 2 min and the supernatant containing the DNA was transferred (without carrying over silica) to a new sterile tube. A second elution was sometimes done. The eluted DNA was stored at 4° short term and −80° long term.

The skin samples were tiny clippings taken from along the mid ventral line of museum skins of *Rattus* and these were also processed in the ancient DNA lab. The skin fragment was placed in a sterile 2 mL tube containing 200 µL of a modified STE buffer (100 mM Tris HCl pH 8.0, 100 mM NaCl and 1 mM EDTA pH 8.0) [32], 60 µL proteinase K (20 mg/mL), 20 µL 1 M dithiothreitol (DTT) and 20 µL of triton X-100 and further macerated in the tube with sterile scissors. The sample was incubated with rotation over night at 55°. In the morning 600 µL of DNA binding solution (5 M GuSCN and 25 mM NaCl) and 100 µL of a silica suspension were added to the sample which was incubated with rotation at 37° for three hours. The subsequent steps were the same as those used for the ancient bone method above.

### PCR conditions

Two regions of the mitochondrial genome were amplified, a 585 bp amplicon of D-loop from the 3′ end and including 27 bp of tRNA proline, and either a 750 bp or a 200 bp amplicon of COI. Due to differences in sequencing success and sequence availability in GenBank the data sets for the two gene regions do not represent all the same species, or specimens. Tables S1 and S2 list the gene regions used for all samples.

### Modern samples

The amplification reactions for the modern samples contained 10 mM Tris HCl pH 8.3; 50 mM KCl; 2.5 mM MgCl_2_, forward and reverse primers at 0.5 µM each; dNTPs at 0.15 mM each; 0.5 U of *Taq* polymerase; 1 µL of DNA template. The primers used to amplify the D-loop region were EGL4L and RJ3R and for the COI region were BatL5310 and R6036R [24]. The PCR (polymerase chain reaction) regime was an initial denaturation step of 94° for 2 min; 35 cycles of 94° for 30 s, 60° for 30 s and 72° for 1 min with a final extension step of 72° for 5 min. Amplicons were sequenced in both the forward and the reverse directions.

### Ancient and historic samples

Amplification of the 585 bp of D-loop for the ancient and historic samples was achieved by amplifying a series of four short overlapping fragments. Since degradation of DNA in ancient samples can result in mis-incorporated nucleotides during PCR, the final DNA sequences were determined from a minimum of two independent amplifications and sequenced in each direction from different PCR products [37]. The regions of sequence overlap were also checked for consistency. Only those samples with consistent sequencing results were used in the subsequent phylogenetic analyses. Primers were designed as needed for the different species. See Table 2 for amplicon details and primer sequences and Fig. S1 for approximate positions of the primers for the overlapping fragments. A single short fragment (197 bp) was targeted for COI using the primers R5838F (5′ to 3′cccamtaccaracrcctctmttt) and R6036R (5′ to 3′ acttctgggtgtccaaagaatca). Generic sequences were added as tags to the 5′ termini of the primers used for the shortest amplicons thus enabling more successful direct sequencing [38].

**Table 2:** Primers used to amplify short fragments of the D-loop region.

	Primer		Species						
	Name	Sequence 5′ to 3′	arg	eve	mor	nio	nit	pra	ver
1	EGL4L	ccaccatcaacacccaaag	*(240)	*(250)	*	*	*	*	*
	EGL7H	tgataacacaggtatgtcc	*						
	EGL7.1H	ggtgtatgtctgataacaca		*	*	*	*	*	*
2	Rarg130F	gacattaaacttaaatcaactaaa	*(190)						
	Reve131F	gacataacattcaaactcaac		*(190)					
	Rp15463F	cgtacattaatttcctttcc			*(247)	*		*	*
	Rnit92F	ccaagcatataagcatgtaat					*(229)		
	R15693R	gttgttgatttcacggagg	*	*	*	*	*	*	*
3	R15621F	cctttctcttccatatgact	*(220)		*		*	*	*
	EGL8.1L	gtgttatcagacatacacca		*(252)					
	Rnio230F	catacaccatataatcataaac				*(237)			
	R15840R	ccatcgagatgtcttattta	*	*	*	*	*	*	*
4	R15722F	cgggcccatacaacttgg	*(219)		*				
	R15775F	catctggttcttacttcagg		*(174)		*	*	*	*
	RJ3R	catgccttgacggctatgttg	*	*	*	*	*	*	*

The four overlapping fragments are indicated by the numbers in the first column. Species names are abbreviated as follows: *R. argentiventer* (arg), *R. everetti* (eve), *R. mordax* (mor), *R. niobe* (nio), *R. nitidus* (nit), *R. praetor* (pra), and *R. verecundus* (ver).

*indicates the primer pairs for each fragment within each species column. The amplicon length (bp before trimming the primer) is indicated by the number in brackets after the forward primer the first time each pair occurs in the table. Primer names ending in L or F are forward primers and H or R are reverse primers. Fig. S1 shows the approximate positions of these primers.

The amplification reactions contained10 mM Tris HCl pH 8.3; 50 mM KCl; 2.5 mM MgCl_2_; BSA 1 mg/mL, forward and reverse primers at 0.5 µM each; dNTPs at 0.15 mM each; 1.0 U of *Taq* polymerase; 3 µL of DNA template. The PCR regime for all primer combinations was an initial denaturation step of 94° for 2 min; 10 cycles of 94° for 20 s, 54° for 20 s and 72° for 20 s followed by 35 cycles of 94° for 20 s, 50° for 20 s and 72° for 20 s with a final extension step of 72° for 5 min.

### Sequencing and alignment

All PCR products were visualised, and subsequently quantified using a low mass ladder for comparison, on ethidium bromide stained 1% agarose gels for the longer fragments or 2% agarose gels (1∶1, agarose: low melt agarose) for the shorter fragments. PCR products were purified either in sephacryl columns (Microspin S300 from Amersham Biosciences), or by enzymatic treatment using ExoSAP-IT from Affymetrix, Inc. Sequencing was carried out at the Massey University Genome Service, Palmerston North, New Zealand, using the BigDye Terminator version 3 sequencing kit, the GeneAmp PCR System 9700 and a capillary ABI3730 DNA analyser, all from Applied Biosystems.

**Figure 2:**
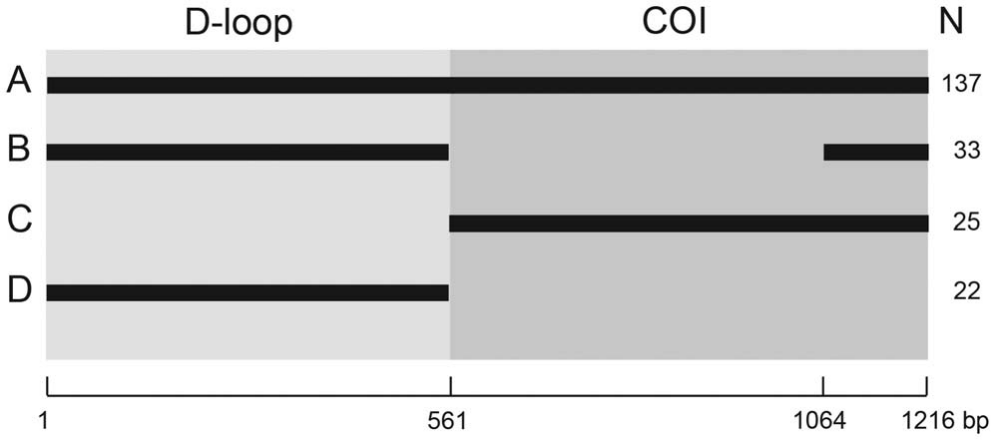
Sequence coverage in the dataset. A total of 217 samples are represented with an aligned sequence length of up to 1216**A** represents samples with the full 1216 bp of D-loop and COI. **B** represents the museum samples with D-loop and a 152 bp fragment of COI sequence. This 152 bp fragment falls at the 5′ end but within the 655 bp COI amplicon. **C** represents samples with only COI sequences and **D** represents samples with only D-loop sequences.

The software package SEQUENCHER (GeneCodes) was used to trim and edit the raw sequences and a consensus sequence was built for each sample. These sequences together with those from GenBank were aligned using ClustalW, as implemented in Geneious version 6.1.3 (created by Biomatters http://www.geneious.com/), and edited by eye within Geneious. The sequences were adjusted to a common length of 544 bp for D-loop and either 655 bp or 152 bp for COI. Sequences from GenBank that did not give complete coverage of these regions were removed from the alignments. Fig. 2 gives an overview of the sequence coverage in the dataset. Samples were sorted into four groups, those for which both D-loop and 655 bp COI sequences were obtained (A), those for which both D-loop and 153 bp COI sequences were obtained (B), those having just 655 bp COI sequences (C), and those having just the D-loop sequences (D). Five alignments were built, one for D-loop, three for COI and one for both regions combined. The D-loop alignment comprised 192 sequences of 544 bp each and, since insertions and deletions (indels) were included, reached a final length of 561 bp (Fig. 2, the D-loop component of sample groups A, B and D). Indels were not coded as binary characters in a separate partition. The COI-655 alignment comprised 162 samples with sequence lengths of 655 bp (Fig. 2, the COI component of sample groups A and C). The COI-655&152 alignment of 195 samples combined all the COI sequences shown in Fig. 2 (the COI component of sample groups A, B and C). The COI-152 alignment included all of the samples from groups A, B and C, but they were reduced to a common length of 152 bp. A concatenated alignment of D-loop and COI was built from the combined sequences from all 217 samples.

### Phylogenetic analysis

We used Bayesian inference analysis (MrBayes version 3.2 [39]) and maximum likelihood analysis (PHYML version 3, [40] and RAxML version 8.0 [41]) to infer phylogenetic relationships among the samples. The models of evolution used were GTR+I+G for D-loop and HKY+I+G for COI as selected in jModelTest 0.1.1 [42]. In the case of RAxML, however, due to the unavailability of the HKY model GTR was used for both regions.

Phylogenies were inferred for the D-loop and the three COI datasets with both PHYML and RAxML. The parameters for PHYML were determined with jModelTest. Bootstrap support under similar substitution models was compared using two implementations of maximum likelihood tree selection criteria (PHYML and RAxML). For the faster, but more model constrained heuristic (RAxML program) we computed 1000 bootstraps unless convergence for nodal support occurred earlier. In the PHYML analyses we made 200–300 pseudoreplicates, which were sufficient to obtain 90–100% support for many clades and produced similar results to those obtained in the RAxML analyses. In the RAxML analyses we partitioned the combined dataset into D-loop and COI to account for positional heterogeneity in the substitution process. Such analyses are not possible with PhyML.

Each MrBayes analysis for D-loop and the COI-655 datasets was run on 4 chains (temperature  = 0.2) for 12 million generations with trees sampled every 1000 generations. As determined in TRACER version 1.5 [43] the first 10% was discarded as burnin, the effective sampling size of all parameters was ≥500 and the potential scale reduction factor approached 1. MrBayes analyses for the other three datasets, two with significant amounts of missing data (concatenated D-loop+COI and COI-655&152), and a third with short sequence length (COI-152), failed to reach convergence after 250 million generations.

## Results

**Figure 3:**
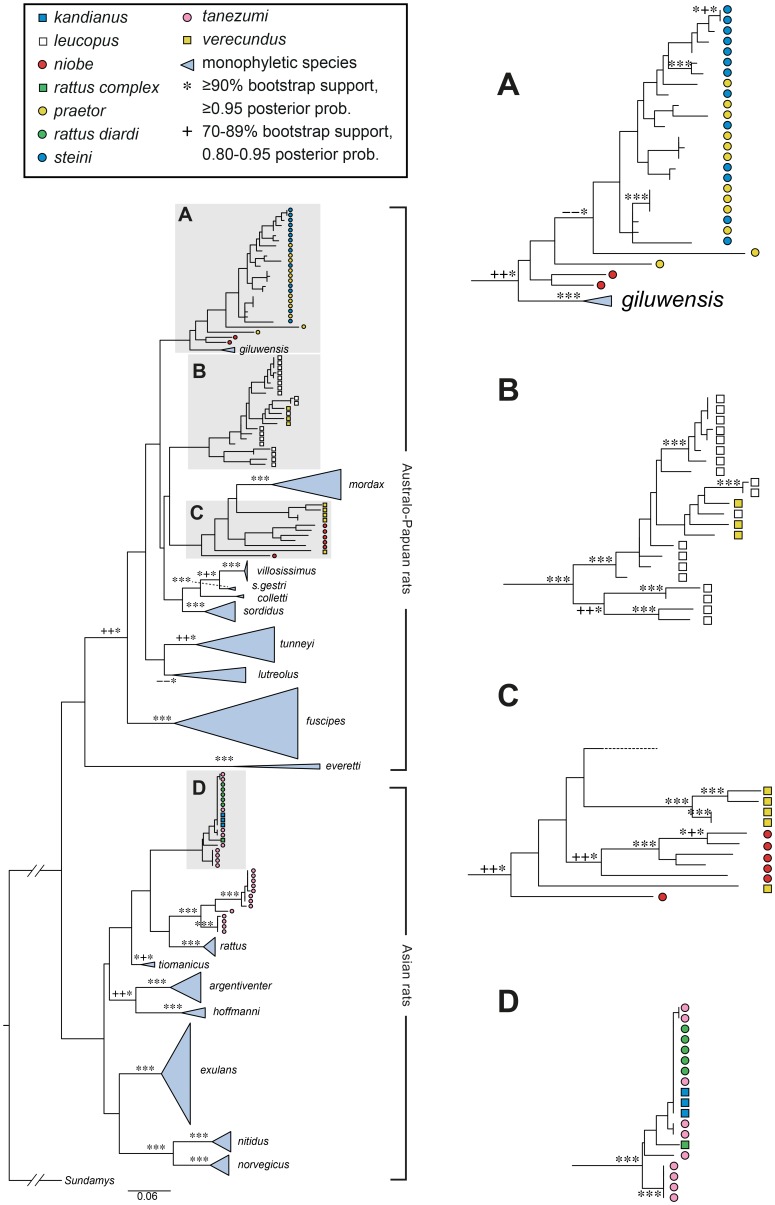
ML tree for D-loop based on 192 taxa with sequence lengths of 560 bp. In this figure and in Figures 4, 5, and 6, nominal species names are used and monophyletic species are indicated by blue triangles that are named on the tree, while polyphyletic or paraphyletic species are colour coded as indicated in the key. The boxes labelled A, B, C, and D are shown enlarged on the right of the figure and, as discussed in the text, they are used in this and subsequent figures to emphasise changes in relative positions and species make-up of clusters. Bootstrap support of ≥70% and Bayesian posterior probabilities ≥0.80 are shown as symbols in the order RAxML/PHYML/MrBayes.

The reassessment of the vouchers held in Sydney at the Australian Museum resulted in ten changes to species identifications of Papua New Guinean rats (see Table 1). Although these ten corrections were based on morphology, they were subsequently found to be consistent with the DNA results. The revised identifications now show that, unlike in previous analyses [24], *R. exulans*, *R. sordidus gestri* and *R. rattus* are all represented by monospecific clades. Further, a clade that previously comprised six nominal species, is now shown to comprise only two species, *R. praetor* and *R. steini* (Fig. 3).

All the longer sequences acquired for this study were deposited in GenBank and their accession numbers are listed in Table S1. GenBank will no longer accept sequences shorter than 200 bp so our 152 bp COI sequences are available in fasta format in the supporting information (File S1).

**Figure 4:**
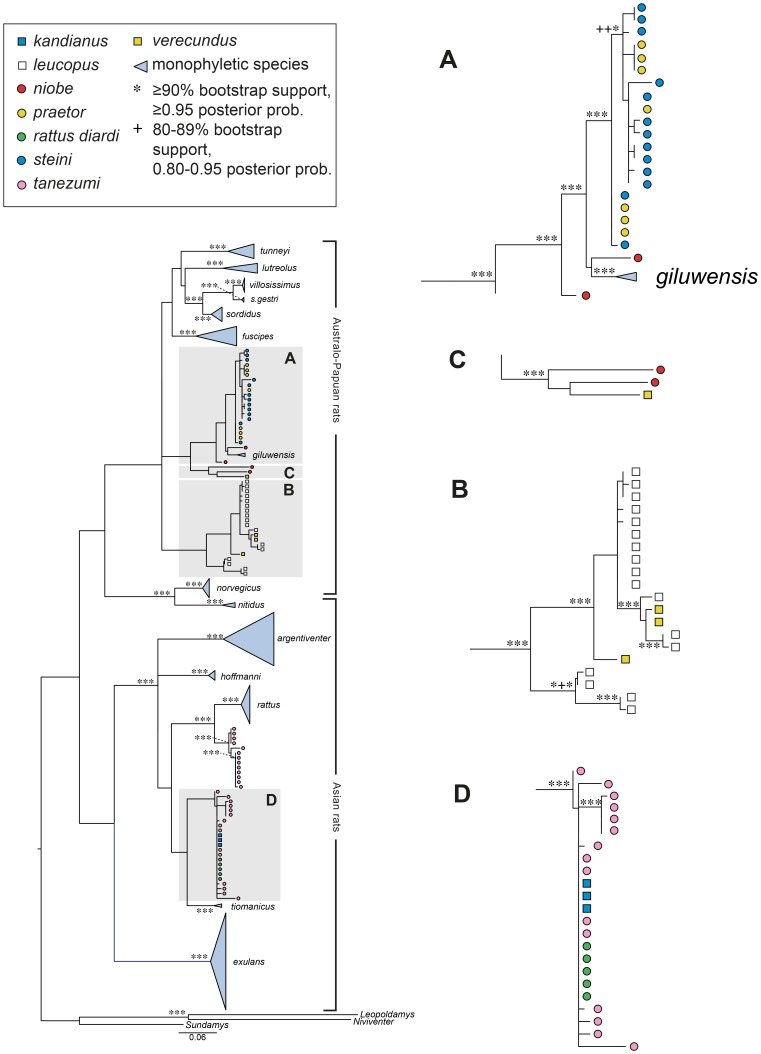
ML tree for COI based on 162 taxa with sequence lengths of 655 bp. As explained in the caption for Fig. 3, monophyletic species are named on the tree while other species are colour coded. Note the changed positions and species make-up shown in the boxes and see further discussion of this in the text. Bootstrap support of ≥70% and Bayesian posterior probabilities ≥0.80 are shown as symbols in the order RAxML/PHYML/MrBayes.

**Figure 5:**
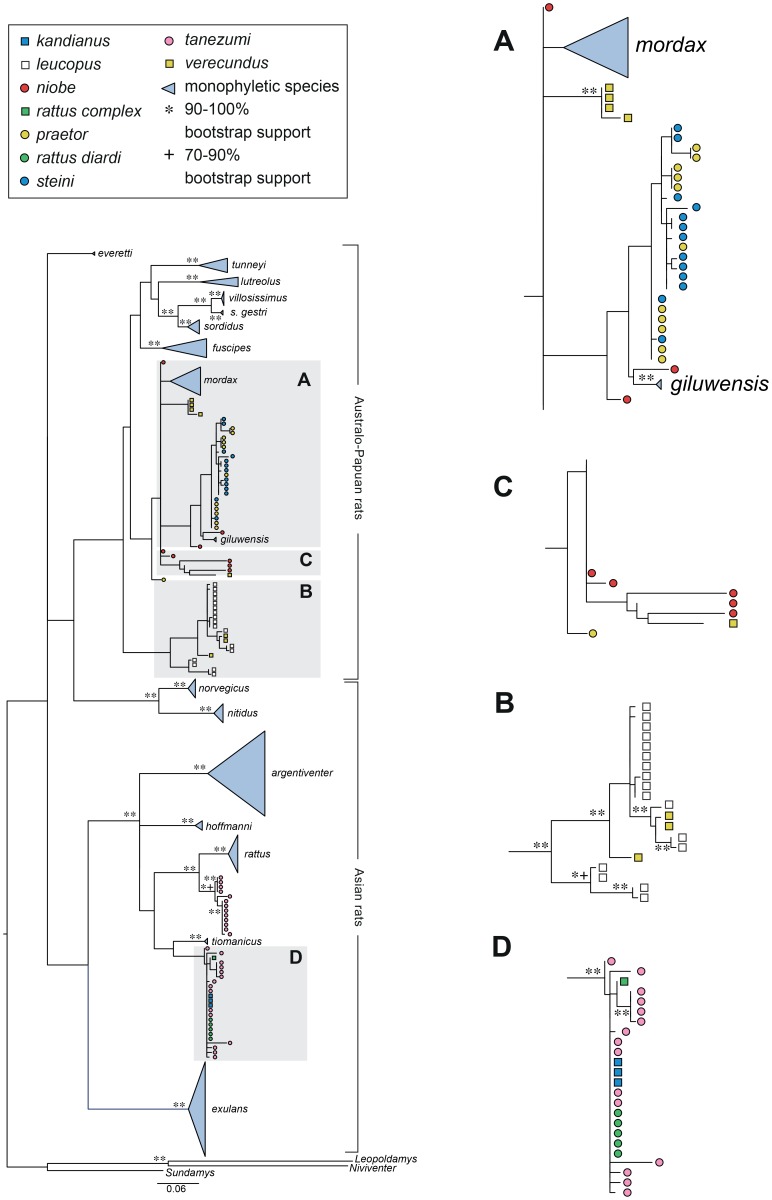
ML tree for COI based on 195 taxa with sequence lengths of either 655 bp. As explained in the caption for Fig. 3, monophyletic species are named on the tree while other species are colour coded. Note the changed positions and species make-up shown in the boxes and see further discussion of this in the text. Bootstrap support of ≥70% is shown as symbols in the order RAxML/PHYML.

**Figure 6:**
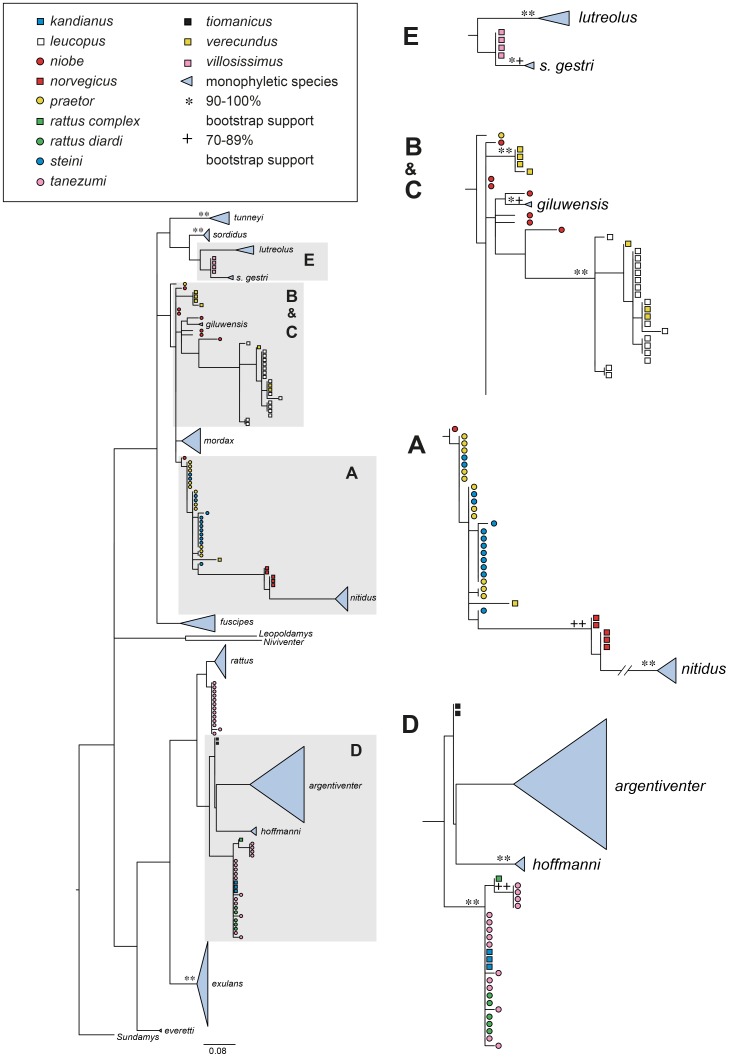
ML tree for COI based on 195 taxa with sequence lengths of 152 bp. As explained in the caption for Fig. 3, monophyletic species are named on the tree while other species are colour coded. Note the changed positions and species make-up shown in the boxes and see further discussion of this in the text. Bootstrap support of ≥70% is shown as symbols in the order RAxML/PHYML.

Of the 40 ancient and historical museum samples, 35 (88%) were successfully amplified for the 544 bp D-loop region, and 33 (83%) for the 152 bp COI region. All of the recently collected New Guinean tissue samples were successfully amplified for both D-loop and the 655 bp COI region. We present four phylogenetic trees in the main body of the paper; Fig. 3 the D-loop tree, Fig. 4 the COI-655 tree, Fig. 5 the COI-655&152 tree and Fig. 6 the COI-152 tree. For future taxonomic purposes, we also present the same trees with full sample identification in supporting information (Figs. S2 to S5). A fifth tree, a combined D-loop and COI analysis, is presented in full in supporting information (Fig. S6).

The maximum likelihood and Bayesian analyses of the D-loop region returned very similar trees. There were minor differences within clades as to the exact placement of individual samples at the tips and there were very slight variations in the backbone. Because the trees were almost identical we show only the PHYML tree, although the bootstrap support from both the RAxML and the PHYML analyses and the Bayesian posterior probabilities are shown for the main nodes common to the three analyses. The D-loop tree (Fig. 3 and Fig. S2) has 17 monospecific clades, although the Australian species *R. colletti* is represented by only a single sample. This tree shows the expected Asian and Australo-Papuan clades as previously reported [24], [27] and has the Philippine endemic *R. everetti* basal in the Australo-Papuan clade although this position is poorly supported (Fig. S2). Within the Asian clade, six monospecific sub-clades occur comprising *R. argentiventer*, *R. exulans*, *R. hoffmanni*, *R. rattus* lineage I (*sensu* Aplin et al. [9]), *R. norvegicus* and *R. rattus* lineage VI (*sensu* Aplin et al. [9]) which is the equivalent of *R. tiomanicus*. One multi-species cluster occurs within the Asian clade (Box D, Fig. 3) comprising samples nominally called *R. tanezumi*, *R. rattus diardi*, *R. kandianus* and *R. rattus* Complex. Also within the Asian clade there is a monospecific group comprising *R. rattus* lineage II (*sensu* Aplin et al. [9]) which is broadly equivalent to *R. tanezumi*.

The Australo-Papuan clade in Fig. 3 is more complex and contains more mismatches, especially among the New Guinean groups. Five Australian *Rattus* species occur in well supported monospecific clades: *R. fuscipes, R. lutreolus, R. sordidus*, *R. tunneyi*, and *R. villosissimus*. The single specimen of *R. colletti* is within a cluster that also includes the Australian *R. villosissimus* and the New Guinean *R. sordidus gestri* but its position lacks support. Three well defined subspecies occur within the ‘*R. fuscipes* species group’; *R. fuscipes fuscipes*, *R. fuscipes coracious* and *R. fuscipes assimilis*. Three *R. fuscipes assimilis* samples from New South Wales cluster together but one sample of putative *R. fuscipes assimilis* falls outside this group and is sister to a single *R. fuscipes greyi* from South Australia (see Fig. S2). The *R. tunneyi* clade comprises two sub-clades that contain representatives of *R. tunneyi culmorum* and *R. tunneyi tunneyi* respectively. Although it has low support, the *R. lutreolus* clade includes samples identified as the subspecies, *R. lutreolus lutreolus* and *R. lutreolus velutinus*. The ‘*R. sordidus* species group’ comprises four well supported clades of *R. sordidus sordidus*, *R. colletti*, *R. sordidus gestri* and *R. villosissimus*. The *R. sordidus gestri* samples had previously been misidentified as *R. rattus* and were the only New Guinean samples found to fall inside a clade of Australian samples, although this is perhaps unsurprising as members of this group are thought to have crossed on land bridges between New Guinea and Australia during times of lowered sea levels. It is notable, however, that the two ‘subspecies’ of *R. sordidus* fail to associate on the D-loop tree.

Of the New Guinean samples those nominally identified as *R. mordax*, *R. sordidus gestri* and *R. giluwensis* are the only samples that occur in monospecific clades. The internal structure of the clade in Box A, Fig. 3 is poorly resolved and comprises members of four nominal species; *R. praetor*, *R. steini*, *R. niobe* and the well supported *R. giluwensis* clade. Box B in Fig. 3 contains representatives of two nominal species; three *R.* cf. *verecundus* samples and 19 *R. leucopus* samples. Four Australian *R. leucopus* samples cluster together and are sister to the 18 New Guinean samples which include both *R. leucopus* and *R.* cf. *verecundus*. The subtree shown in Box C, Fig. 3 contains a monospecific clade of *R. mordax* and well supported clades of *R. niobe* and *R. verecundus*, containing most but not all of their respective samples. The two outlying samples of *R. niobe* and *R. verecundus* are from different localities to samples in the main clusters.

The phylogeny seen for the COI-655 analysis (Fig. 4 and Fig. S3) is very similar to that inferred for the D-loop. The sample base is not identical (see Fig. 2, Tables S1 and S2) but the phylogenies share 14 monospecific clades. Unlike the sequence availability for D-loop, there were no 655 bp long sequences of COI available from *R. colletti, R. mordax* or *R. everetti*. The split between the Asian and Australo-Papuan rats is equivocal in the COI phylogenies with *R. norvegicus* and *R. nitidus* basal in the Australo-Papuan clade instead of in the Asian clade, albeit with low support.

Analysis of the combined long and short COI sequences (COI-655&152 phylogeny Fig. 5 and Fig. S4), returned 16 monospecific clades. These are essentially the same species clades as seen in the D-loop phylogeny (Fig. 3) except for the absence of *R. colletti* and the presence of only a single *R. everetti* sample. Although the branching patterns vary between the trees, the same samples generally cluster together across these two phylogenies. The COI-152 phylogeny (Fig. 6 and Fig S5) has much poorer resolution than the COI-655&152 phylogeny (Fig. 5). It resolved only 13 monophyletic species, *R. niobe* samples have become more widespread in the tree and two of the three outgroup samples (*Leopoldamys* and *Niviventer*) have become displaced. The same samples were included in both phylogenies but all the sequences used to infer the Fig. 6 phylogeny were short, whereas the Fig. 5 phylogeny had a mixture of long and short sequences.

The combined D-loop and COI analysis tree shown in Fig S6 has a similar topology to the separate D-loop and the COI-655 trees (Fig. 3 and Fig. 4 respectively). There is good support for the same 17 monospecific clades as seen in the D-loop tree (Fig. 3). Nodal support levels are similar across the single gene regions and the combined gene region trees (Fig S6) and are comparable to those present in the D-loop trees under RAxML, PHYML and MrBayes (Fig. 3). Nodes with moderate to high support found on both the D-loop tree and the combined D-loop and COI tree are indicated in Fig. S6. Table 3 compares the level of support for monophyletic species over 11 phylogenies. Of the 25 nominal species, eleven species were monophyletic in all analyses (i.e. *R. argentiventer, R. exulans, R. fuscipes, R. giluwensis, R. hoffmanni, R. lutreolus, R. nitidus, R. sordidus, R. sordidus gestri, R. tunneyi*, and *R. villosissimus*). Three species were monophyletic in all phylogenies except in the COI-152 phylogeny (i.e. *R. norvegicus*, *R. tiomanicus* and *R. villosissimus*). Two species were monophyletic for the D-loop and the combined analyses but there were no 655 bp COI sequences available for these samples (i.e. *R. everetti* and *R. mordax*). The single specimen of *R. colletti* is not found within any other species clade and is represented by only a D-loop sequence. Eight species were never monophyletic in any analyses (i.e. *R. kandianus, R. leucopus, R. praetor, R. niobe, R. rattus diardi, R. steini, R. tanezumi* and *R. verecundus*).

**Table 3:** The presence of monophyletic species across the trees compared with nominal species designations.

	Dloop+COI	D-loop			COI-655			COI-655&152		COI-152	
Nominal Species	R	R	P	B	R	P	B	R	P	R	P
*R. argentiventer*	M	M	M	M	M	M	M	M	M	l	l
*R. colletti*	s	s	s	s	-	-	-	-	-	-	-
*R. everetti*	M	M	M	M	-	-	-	s	s	s	s
*R. exulans*	M	M	M	M	M	M	M	M	M	M	M
*R. fuscipes*	M	M	M	M	M	M	M	M	M	l	l
*R. giluwensis*	M	M	M	M	M	M	M	M	M	M	m
*R. hoffmanni*	M	M	M	M	M	M	M	M	M	M	M
*R. kandianus*	x	x	x	x	x	x	x	x	x	x	x
*R. leucopus*	x	x	x	x	x	x	x	x	x	x	x
*R. lutreolus*	M	l	l	M	M	M	M	M	M	M	M
*R. mordax*	M	M	M	M	-	-	-	l	l	l	l
*R. niobe*	x	x	x	x	x	x	x	x	x	x	x
*R. nitidus*	M	M	M	M	M	M	M	M	M	M	M
*R. norvegicus*	M	M	M	M	M	M	M	M	M	x	x
*R. praetor*	x	x	x	x	x	x	x	x	x	x	x
*R. rattus*	M	M	M	M	M	M	M	M	M	l	l
*R. r. diardi*	x	x	x	x	x	x	x	x	x	x	x
*R. sordidus*	M	M	M	M	M	M	M	M	M	M	M
*R. s. gestri*	M	M	M	M	M	M	M	M	M	M	m
*R. steini*	x	x	x	x	x	x	x	x	x	x	x
*R. tanezumi*	x	x	x	x	x	x	x	x	x	x	x
*R. tiomanicus*	M	m	M	M	M	M	M	M	M	x	x
*R. tunneyi*	M	m	m	M	M	M	M	M	M	M	M
*R. verecundus*	x	x	x	x	x	x	x	x	x	x	x
*R. villosissimus*	M	M	M	M	M	M	M	M	M	x	x

The tree estimation methods are represented as: R = RAxML; P = PHYML; B = MrBayes. The evidence for monophyly is represented as: M =  monophyletic species with good support (≥90% bootstrap; ≥0.95 posterior probability); m =  monophyletic species with moderate support (70–90% bootstrap; 0.80–0.95 posterior probability); l =  monophyletic species with low support; x =  non-monophyletic species; -  = species not present in tree; s =  single sample.

## Discussion

### Molecular taxonomy of *Rattus* species

Compared with the earlier study of Robins et al. [24], the current analysis contains fewer instances of mismatch between traditional morphology-based and molecular-based taxonomic identifications of *Rattus*, especially in the case of the New Guinean species. In large part, this reflects the particular effort taken to obtain sequences from specimens that either were used in the previous taxonomic revision of Taylor et al. [5] or were available for confirmatory morphological examination by Aplin and Robins. Despite this fact, a number of incongruences remain, particularly for several groups of Asian and New Guinean rats.

For the Asian rats, our sampling includes nine well supported clades. Only one of these, evident in Box D in Figs. 3 to 6, contains multiple nominal species. The genetic signature and geographic origin (Java, Malaysia, Sri Lanka, Sulawesi and Vietnam) of this cluster of samples identifies it as *R. rattus* Complex (RrC) Lineage IV of Aplin et al. [9] (equivalent to lineage R3 of Pagès et al. [23]). The variety of names still in use in museums and tissue collections for rats of this clade reflects the persistent local use of taxonomic names for local black rat variant populations (e.g. *R. kandianus* from upland Sri Lanka, *R. rattus diardi* for the Malay Peninsula and western Sundaic islands). At least some of these rats were introduced in prehistoric to early historic times [9]. Moreover, in the particular case of Lineage IV of the RrC, the name variation reflects continued uncertainty over whether or not this mitochondrial lineage warrants recognition as a distinct taxonomic entity in the face of growing evidence for widespread nuclear introgression between Lineages II and IV [44]. Broader genomic analyses currently underway by Aplin and colleagues will throw much needed light on this issue.

Included within the RrC Lineage IV clade are a number of Vietnamese rat sequences published by Balakirev and Rozhnov [30]. For reasons that are not at all clear, these were interpreted as the first Indochinese representatives of *R. tiomanicus* (included in RrC Lineage VI of Aplin et al. [9]). This conclusion is almost certainly erroneous and is further confounded by the fact that their sampling included true *R. tiomanicus* from the Malay Peninsula which they labelled *Rattus* sp. and discussed as a possible new taxon. Taxonomic reallocation of samples without examination of voucher specimens must always be undertaken with some caution; however, in this case we feel justified in amending ‘*R. tiomanicus*’ of Balakirev and Rozhnov [30] to RrC Lineage IV of Aplin et al. [9] and ‘*Rattus* sp.’ of Balakirev and Rozhnov [30] to *R. tiomanicus*.

For most of the Australian native *Rattus*, the mitochondrial phylogenies show good taxon resolution and, for two of these, the resolution is good down to the subspecies level; in the case of *R. fuscipes* (three of four subspecies) and *R. tunneyi* (two subspecies) (see Fig. S2 and S4). The shared Australo-Papuan species, *R. leucopus*, also shows good taxon resolution down to subspecies level, albeit with some qualification in regard to the New Guinean populations (see below).

For both *R. fuscipes* and *R. sordidus* the intraspecific clade structure shows partial disagreement with current taxonomy. Within *R. fuscipes*, the available sequences of *R. fuscipes assimilis* and *R. fuscipes greyi* from Eastern Australia do not assort according to currently recognised subspecies. Broader geographic and genomic sampling within *R. fuscipes* is needed to resolve this mismatch.

Our taxon coverage for *R. sordidus* is expanded over previous studies through the inclusion of *R. sordidus gestri*, an endemic of the southeast peninsula of New Guinea [5]. This taxon is morphometrically and chromosomally distinct from typical *R. sordidus sordidus* of northeast Australia and from *R. sordidus aramia* of the Trans-Fly region of southern New Guinea [5], [45], [46]. In our analysis *R. sordidus gestri* clusters with *R. colletti* and *R. villosissimus*, with typical *R. sordidus* identified as the sister to this clade. While formal taxonomic change is premature, our finding is further indication that *gestri* may represent a full species within the ‘*R. sordidus* species group’.

All of the *R. leucopus* samples fall into a single well supported clade (Box B, Fig. 3–6) which shows strong phylogeographic structure. The primary division is between the four Australian samples and the more extensively sampled New Guinean population. As reported previously by Rowe et al. [26], the Australian samples are divided again into southern and northern clades, corresponding to the recognized subspecies *leucopus* and *cooktownensis*, respectively [46]. These allopatric populations show fixed chromosomal rearrangements [47], allozymic differences [48], and reciprocal monophyly on Rowe et al.'s [26] multi-gene trees but they show weak morphological differentiation [46].

Our expanded sampling of New Guinean populations derives largely from localities in the upper and middle catchment of the Purari River in Gulf Province, but includes one sample from Oro Province on the southeast peninsula (see Fig. 1). The Oro sample is presumably attributable to the subspecies *R. leucopus dobodurae* [5] but the series from Gulf Province is geographically and morphologically intermediate between this taxon and *R. leucopus ringens* which occurs further to the west in the southern lowlands. Further west again, this form is replaced by *R. leucopus ratticolor* [5] which remains unsampled genetically. Rowe et al. [26] reported a possible clade distinction between the Oro and Gulf Province populations but this disappears with our larger sampling of the population in Gulf Province. If these populations are representative of *R. leucopus dobodurae* and *R. leucopus ringens* respectively, as might be inferred on biogeographic criteria, then our findings would indicate incomplete mitochondrial lineage sorting. An intriguing finding of this study is the inclusion within the *R. leucopus* clade of three samples attributed to *R*. cf. *verecundus* from localities in Chimbu and Southern Highlands Provinces (see Fig. 1). This anomaly will be discussed further below.

Among the remaining exclusively New Guinean species, only *R. giluwensis* and *R. mordax* are represented by simple monotypic clusters on the phylogenies. All other nominal species either appear in at least two clades on the trees or form polytypic clades. This remains true even after a few cases of mismatch between taxonomic identity and clade membership were resolved through re-examination of voucher specimens (see Table 1). The possible reason for each of these cases of clade mismatch is discussed in the following section.

### Phylogenetic discordance of New Guinean *Rattus*

Our expanded sampling of New Guinean *Rattus* exposed a number of persistent (i.e. not resolved by voucher reassessment) mismatches between nominal taxon and molecular clade. In brief these are:

1. Placement of nominal *R.* cf. *verecundus* within a clade otherwise comprising New Guinean samples of *R. leucopus*;
2. Inclusion within a single clade of numerous samples of nominal *R. steini* and nominal *R. praetor*;
3. Placement of nominal *R. niobe* and nominal *R. verecundus* in multiple locations within the tree structure.

Each of these apparent anomalies warrants further discussion of the underlying causes.

Our three samples of *R*. cf. *verecundus* come from three different localities in the foothills of southern New Guinea and are representative of larger regional series with the same morphology. In body size and general cranial morphology they resemble *R. verecundus* [5] but they differ from all regional forms of this species in having much harsher fur with numerous spines, more akin to the pelage of *R. leucopus*. The samples of *R*. cf. *verecundus* yielded three different mitochondrial haplotypes which cluster with another three haplotypes derived from middle Purari River samples of *R. leucopus* subsp. As will be discussed further below, other samples of *R. verecundus* fall in several places on the gene trees but are all well outside the *R. leucopus* clade.

There are several plausible interpretations of the position of *R*. cf. *verecundus* on the mitochondrial phylogeny. One is that the ‘taxon’ comprises hybrids between *R. leucopus* and a small-bodied species of *Rattus*, potentially some form of *R. verecundus* or *R. niobe*. However, it is relevant to note that typical examples of *R. verecundus* were not obtained at any of the same localities and examples of *R. niobe* were obtained in proximity to only one of the three sites (Bobole in Southern Highlands Province). Moreover, at two of the three localities (Noru in Chimbu Province and Bobole in Southern Highlands Province), no examples of typical *R. leucopus* were obtained at the same altitude although they were found regionally at lower elevations. For these reasons, immediate hybrid origin can probably be ruled out, leaving three alternatives: 1. the populations represent a regional form of *R. verecundus* that has experienced past hybrid activity with *R. leucopus* and which now carries an introgressed mitochondrial genome of *R. leucopus*; 2. the populations represent a distinct taxon that is morphologically convergent on *R. verecundus* and has arisen very recently by cladogenesis from within the regional population of *R. leucopus*; and 3. the populations represent a distinct taxon that has originated through hybridization between *R. leucopus* and a second parental lineage, as yet unidentified. It is relevant to note in this context that the karyotype of *R. leucopus dobodurae* (2N = 34) differs from that of all examples of *R. niobe* and *R. verecundus* (with 2N = 32) investigated to date [45] but perhaps not in any way that would negate production of viable F1 offspring and backcrosses. Further study of this fascinating regional population is clearly needed to clarify its taxonomic status and mode of origin.

Box A on Figs. 3–6 contains all samples of *R. praetor* and *R. steini* and is the equivalent of clade PNGI in Robins et al. [24], though it now includes two rather than six nominal species after the correction of some voucher misidentifications. Notably, this includes samples reported by Rowe et al. [26] as *R. novaeguineae* which are now allocated on morphological criteria to *R steini* (Table 1). As shown on Figs. 3–6, samples of *R. praetor* and *R*. *steini* fail to segregate on either D-loop or COI phylogenies although the two most basal samples, each on relatively long branches, are both derived from samples of *R. praetor*. Taylor et al [5] distinguished two subspecies of *R. praetor*: *R. praetor coenorum* on mainland New Guinea and *R. praetor praetor* on the islands east of New Guinea through to the Solomon Islands. Our sampling includes one sample from each of New Ireland (PrPN_580077) and Bougainville Island (PrPN_277061). These derived haplotypes are similar (1.5% divergence) and form a terminal cluster on the phylogenies; however, this cluster is embedded within the wider diversity of *R. praetor* hence the *praetor* versus *coenorum* distinction is not strongly supported by our analyses. A hint of alternative phylogeographic structure within *R. praetor* is seen in the fact that two of our samples from western New Guinea (PrIJ_277021 from Sansapor and PrIJ_295120 from Jayapura) produced the two most divergent haplotypes (7.7% divergence) within this clade. Morphological assessment of *R. praetor* from western New Guinea by Aplin and Helgen (unpublished) also points to the possibility of taxonomic complexity within mainland *R. praetor*. Further work is needed on this interesting group, including wider sampling to identify the likely source area of introduced populations on the eastern Melanesian islands.

*Rattus steini* is morphologically well-differentiated from *R. praetor* [5] and the inter-digitation of the two species on the mitochondrial phylogenies cannot be explained by misidentifications. The morphological differences are best illustrated by the series of specimens from Munbil, in the Victor Emmanuel Range of West Sepik Province, where the two species are sympatric in garden and regrowth habitats at around 900–1000 m above sea level. The large voucher series held by the Australian Museum from Munbil is readily divisible into two species on the basis of body size (*praetor* reaches a much larger adult size) and foot proportions (longer and broader in *praetor*) and there are no obvious intermediate morphologies. Although there is a suggestion of east to west phylogeographic structure in the combined sample of *R praetor* and *R. steini* the overall pattern in the phylogenies suggests either mitochondrial introgression caused by low frequency hybridization or incomplete sorting of mitochondria between two recently separated species. This problem was not evident in the Rowe et al. [26] phylogeny as they had fewer samples (three *R. praetor*, one *R. steini* and four nominal *R. novaeguineae* which we consider to be *R. steini*, compared with our twelve *R. praetor* and seven *R. steini*).

Sequences from our *R. niobe* samples fall in three different places in the D-loop phylogeny making the nominal taxon deeply polyphyletic (Fig. 3). One cluster of two specimens from each of Chimbu and Southern Highlands Province is placed sister to the *steini/praetor* clade. Another clade consists of a series of historical vouchers from the Wau area of Morobe Province, with one individual (NiPN075) from Mt Albert Edward in the Owen Stanley Range of Central Province as a sister lineage. Finally, one specimen (NiPN077) from West Sepik Province is placed as a sister lineage to a polytypic clade that includes *R. mordax*, some *R. verecundus* and the Wau/Mt Albert Edward *R. niobe* specimens. Examination of the vouchers from each series revealed significant morphological contrasts between each of these populations and the congruence of divergent mitochondrial clades and morphological types is good evidence for the presence of multiple species within Papua New Guinean populations of *R. niobe*. Our geographic sampling of this group, however, is drawn exclusively from the eastern half of New Guinea so our perspective on species diversity most likely remains incomplete. In particular, we may have altogether missed sampling western New Guinean lineages that were grouped by Taylor et al. [5] as *R. niobe arrogans* but more recently split into *R. arrogans*, *R. pococki* and *R. arfakiensis* by Musser and Carleton [1]. However, we leave open the possibility that our sample from Sol River in West Sepik Province (NiPN077) may represent the taxon *R. pococki* as employed by Musser and Carleton [1]. Of the remaining samples, the specimen from Mt Albert Edward is geographically most proximate to the type locality for typical *R. niobe*. However, caution is urged in the allocation of this name to any one of the clades because of the possibility of elevational taxon replacement within single regions, as described for other New Guinean regions by Flannery and Seri [49], Musser and Carleton [1], and Aplin and Kale [50].

Before we can draw any firm conclusions regarding the taxonomic status of *R. niobe*, more genetic sampling with associated morphological assessments is needed over wider geographic regions. In addition, before our tentative conclusion of multiple species within *R. niobe* is taken as fact, further consideration should be given to alternative explanations of the genetic pattern. Clearly, misidentification can be discounted in this case as *R. niobe*, because of its very small adult body size and decidedly ‘shrew-like’ form, is among the most readily identified of all *Rattus* species [5], [29]. However, far less certain is the possible role of past hybridization and introgression in the formation of mitochondrial diversity within *R. niobe*. In this regard we note that each of the various ‘*niobe*’ haplotype clusters is highly divergent not only from one another but also from all other nominal taxa. If introgression did occur between *niobe* and other taxa then it must have occurred either early in the radiation of New Guinean *Rattus* or, if it occurred more recently, then each of the other parental taxa must have become extinct since the time of introgression. These scenarios are possible but seem less parsimonious than the suggestion of cryptic species diversity within *R. niobe*, especially in view of the observed congruence between morphological and genetic patterns of variation within the group.

Nominal *R. verecundus* also occurs in two different places in the phylogenies. In this case the clusters do correspond to morphologically distinctive forms from discrete geographic regions. Taylor et al. [5] actually divided *R. verecundus* into four subspecies, *R. verecundus mollis*, *R. verecundus verecundus*, *R. verecundus unicolor* and *R. verecundus vandeuseni*, subsequently Flannery [29] and Musser and Carleton [1] separated *R. vandeuseni* as a separate species because it occurs in parapatry or possibly even sympatry with the form *R. verecundus verecundus*. On geographic and morphological grounds, our samples appear to represent two of the three subspecies of *R. verecundus*, namely *R. verecundus mollis* [four samples which cluster together in both the D-loop (Box C, Fig. 3) and COI phylogenies (Box A in Fig. 5 and Box B&C in Fig. 6)] and *R. verecundus verecundus* (the single sample which is basal in Box C, Fig. 3). As argued above for *R. niobe*, the most likely explanation for the observed pattern is the presence of at least two species within *R. verecundus*. But while our results strongly suggest that *R. verecundus verecundus* and *R. verecundus mollis* should be recognised as distinct species, we urge further studies including investigation of the forms *unicolor* and *vandeuseni* before any formal changes are advanced. The three samples of *R.* cf. *verecundus* that occur in the *R. leucopus* clade in Box B, Fig. 3 to 6 further complicate the picture for *R. verecundus* and its allies.

The phylogenetic position of *R. everetti*, a Philippines endemic, is intriguing but uncertain. The analysis by Jansa et al. [51] that used cyt *b* and IRBP showed it to be within the same clade as samples of the *Rattus* species *R. praetor*, *R. exulans* and *R. tanezumi*, as well as with *Tarsomys apoensis* and two species of *Limnomys*. Our *R. everetti* sequences clustered together in the D-loop and the combined D-loop and COI analyses and occupy a position within *Rattus* that is basal to the Australo-Papuan clade, although there is low support for this relationship. We were able to amplify only one of the samples for COI and again its position was highly uncertain.

### Potential for phylogenetic species identification

As expected, the D-loop (Fig. 3) and the COI-655&152 (Fig. 5) gene trees provided the greatest information for species identification, with the COI-655 (Fig. 4) gene tree being less informative as it contained fewer species in it. The inferred phylogeny from the combined short and long COI sequences was compatible with the D-loop phylogeny while the gene tree reconstructed from short COI sequences alone (Fig. 6) was considerably less well resolved. Remarkably, much of the primary clade structure including that of the subspecies was nevertheless retained even within the COI-152 gene tree (Fig. S5). One exception concerned the placement of *R. villosissimus*, whose sibling *R. colletti* is not represented in our COI data base. The encouraging results obtained with a data set comprising short and long sequences suggests that the use of short COI sequences for the identification of rats will in future be most successful for phylogeny reconstruction if there are longer reference sequences available to scaffold the short sequences. Our gene trees were similar for analyses of D-loop or COI markers. Phylogenetic resolution was not greatly improved by concatenating the two regions, in our situation where there was missing data. From a purely pragmatic point of view, it is cheaper and easier to sequence one rather than two gene regions and the choice of which region to use may well depend on the size of the reference dataset and the ease of alignment (COI being definitely easier to align). Maximum likelihood methods of tree building were more successful and faster than MrBayes analyses, which failed to converge, presumably due to missing data [52].

Single gene molecular approaches will fail to generate precise taxonomic identifications in groups that contain any one of the following: unrecognised cryptic species, species subject to genetic introgression, or species that show incomplete lineage sorting. Our findings for New Guinean *Rattus* highlight likely instances of all of these confounding phenomena. Similarly, there is mounting evidence for a complex genetic history involving mitochondrial introgression in the case of the Asian black rat group (*Rattus rattus* Complex of Aplin et al. [9], Pagès et al. [23], [44], Conroy et al. [53], and Lack et al. [54]). No such evidence has yet emerged for Australian *Rattus*, although we note that many of the species are capable of fertile interbreeding under laboratory conditions [55], [56]. The possibility of phylogenetic and morphological mismatch among Australian *Rattus* will remain until more comprehensive sampling of morphologically verified samples is undertaken.

Despite these caveats, this study extends and reinforces the usefulness of phylogenetic identification of *Rattus* species using either D-loop or COI sequences although the short COI sequences alone are less informative. In the case of the New Guinean rats, however, there is a pressing need for more extensive genetic and morphological investigation of several groups that appear to harbour instances of cryptic speciation, introgression and/or incomplete lineage sorting.

Issues of phylogenetic discordance and potential introgression raise questions about the timing of divergences of the *Rattus* lineages. A number of authors have estimated the timing of speciation within *Rattus* and the results are reasonably concordant given the variety of molecular methods used, the different genes and species analysed. The most recent common ancestor (tmrca) of *Rattus* was estimated at ∼2.7 Mya using LINE-1, long interspersed repeated retrotransposable elements [57] and whole mitochondrial genomes [27]; slightly younger at ∼2.4 Mya using D-loop and four nuclear genes [26]; and older at ∼3.8 Mya using cytochrome *b* [9]. Also from these studies the divergence of Asian rats into several lineages was estimated at between 2.3 and 1.5 Mya with further radiations from ∼1 to 0.2 Mya. Among the Australo-Papuan rats speciation is estimated to be rapid and extensive with most divergences occurring between ∼1.6 and 0.2 Mya. [26], [27]. These recent, rapid and ongoing speciation events occurring over the last million years or so have doubtless contributed to incomplete lineage sorting suggested in the current work and to the limited morphological variation seen among the lineages.

## Conclusions

By using samples taken from museum specimens included in prior taxonomic revisions and others with recently collected voucher specimens we have been able to resolve some, but not all, of the polyphyletic clades identified in earlier analyses of the genus *Rattus* [24], [26]. The inferred phylogenies from either D-loop or COI, resulting in 17 or 16 single species clades respectively, are both useful and important for future species identification. While the short COI sequences alone are insufficient for reliable identification of some *Rattus* species, they are much more useful when longer reference sequences from the query species are present in the alignment. The New Guinean rats remain the most problematic although the reassessment of vouchers has enabled us to eliminate simple issues of misidentification, and to pinpoint a number of likely cases of cryptic species diversity, genetic introgression and/or incomplete lineage sorting. Each of these cases can now be investigated in detail with combined morphological and genetic studies that will resolve their taxonomy as well as provide new insights into evolutionary processes that have underpinned the remarkable recent radiation of *Rattus* in the Australo-Papuan region.

## Supporting Information

Figure S1 **The relative positions of the D-loop primers, designed to amplify the museum samples, against a generalised** ***Rattus*** **sequence.** Green arrows indicate forward primers and red arrows indicate reverse primers.
(PDF)

Figure S2 **PHYML tree for D-loop.** Based on 192 taxa with sequence lengths of 560 bp. Samples are identified. Nodal support is indicated as in Fig. 3.
(PDF)

Figure S3 **PHYML tree for COI-655.** Based on 162 taxa with sequence lengths of 655 bp. Samples are identified. Nodal support is indicated as in Fig. 4.
(PDF)

Figure S4 **PHYML tree for COI-655&152.** Based on 195 taxa with sequence lengths of either 655 bp or 152 bp. Samples are identified. Nodal support is indicated as in Fig. 5.
(PDF)

Figure S5 **PHYML tree for COI_152.** Based on 195 taxa with sequence lengths of 152 bp. Samples are identified. Nodal support is indicated as in Fig. 6.
(PDF)

Figure S6 **RAxML tree for D-loop+COI.** The combined dataset of all 217 samples including all the D-loop and COI sequences. Samples are identified and bootstrap support from RAxML is shown. In addition the support for nodes also present in the D-Loop tree (Fig. 3 and Fig. S4) is shown. The levels of support are indicated as follows: * = 90–100% bootstrap or ≥0.95 posterior probability, + = 70–89% bootstrap or 0.80–0.95 posterior probability. The symbol order is RAxML combined tree/RAxML D-loop tree/MrBayes D-loop tree.
(PDF)

Table S1 **Sample Information including GenBank accession numbers for newly published sequences used in this study.**
(PDF)

Table S2 **Previously published sequences.**
(PDF)

File S1 **COI-152 bp sequence data in fasta format.**
(TXT)
