# Correction: Phylogenetic Species Identification in *Rattus* Highlights Rapid Radiation and Morphological Similarity of New Guinean Species

**DOI:** 10.1371/journal.pone.0107667

**Published:** 2014-09-02

**Authors:** The PLOS ONE Staff

There are errors to the Accession and Code in Analyses columns of Table S1. Please view the correct Table S1 below.

## Supporting Information

Table S1 **Sample Information including GenBank accession numbers for newly published sequences used in this study.**
(PDF)
