# Supplementary figures and images for: Phylogenetic Species Identification in Rattus Highlights Rapid Radiation and Morphological Similarity of New Guinean Species
Source: PLoS One. 2014 May 27;9(5):e98002. doi: 10.1371/journal.pone.0098002 (PMC4035291; doi:10.1371/journal.pone.0098002)

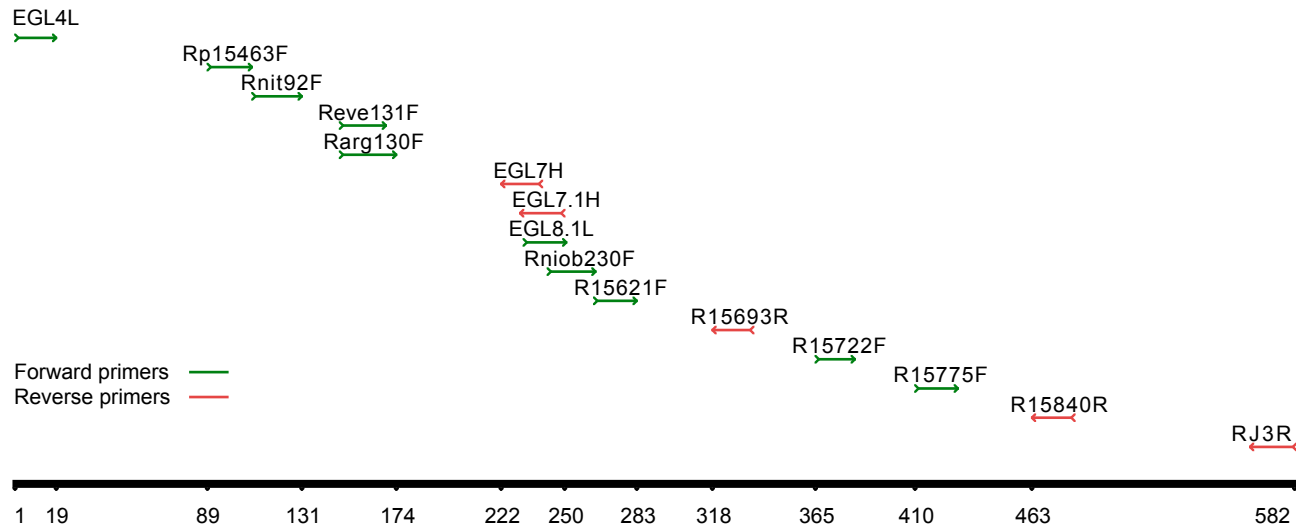

D-loop sequence 582 bp long

Supplement: Figure S1 — The relative positions of the D-loop primers, designed to amplify the museum samples, against a generalised Rattus sequence. Green arrows indicate forward primers and red arrows indicate reverse primers. (PDF) [file pone.0098002.s001.pdf]

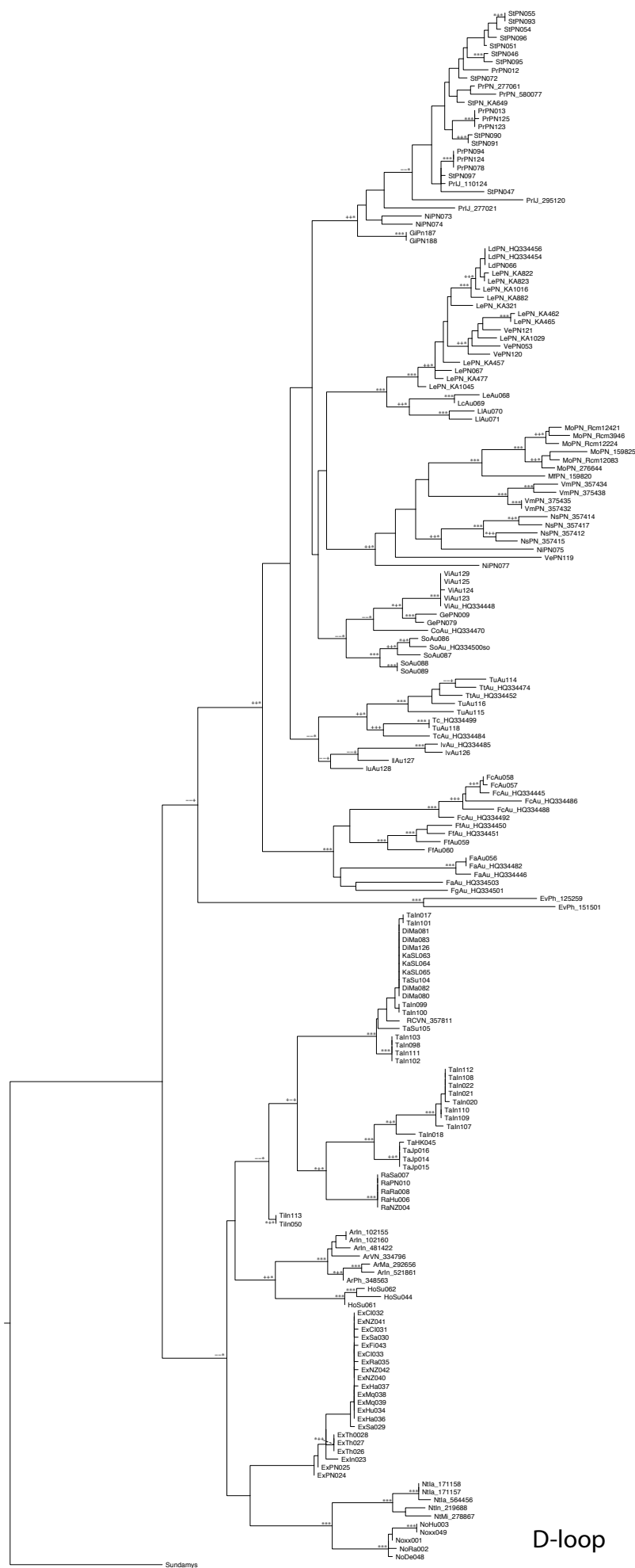

D-loop

Supplement: Figure S2 — PHYML tree for D-loop. Based on 192 taxa with sequence lengths of 560 bp. Samples are identified. Nodal support is indicated as in Fig. 3. (PDF) [file pone.0098002.s002.pdf]

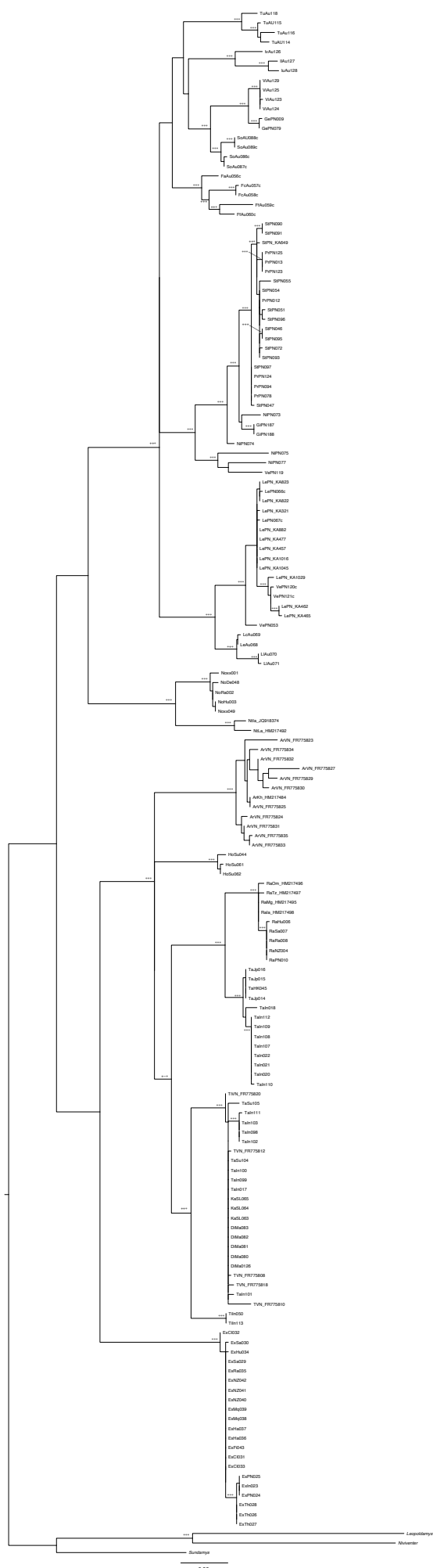

Supplement: Figure S3 — PHYML tree for COI-655. Based on 162 taxa with sequence lengths of 655 bp. Samples are identified. Nodal support is indicated as in Fig. 4. (PDF) [file pone.0098002.s003.pdf]

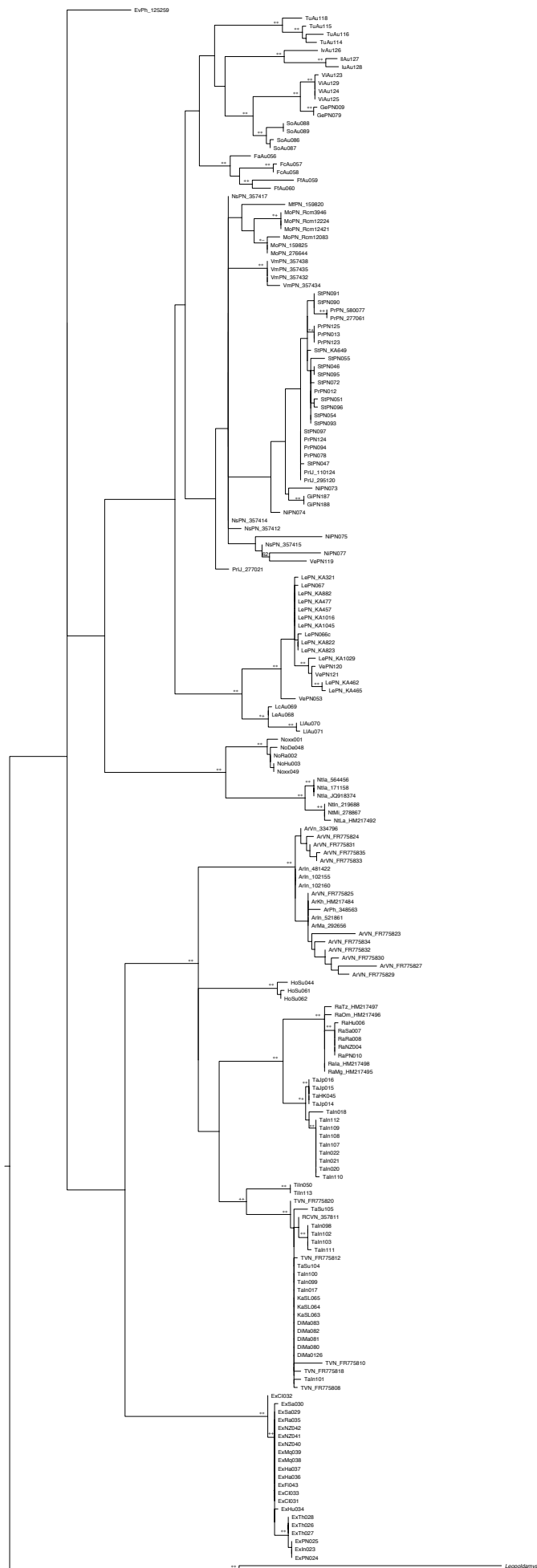

0.06

COI-655 & 152 bp

Supplement: Figure S4 — PHYML tree for COI-655&152. Based on 195 taxa with sequence lengths of either 655 bp or 152 bp. Samples are identified. Nodal support is indicated as in Fig. 5. (PDF) [file pone.0098002.s004.pdf]

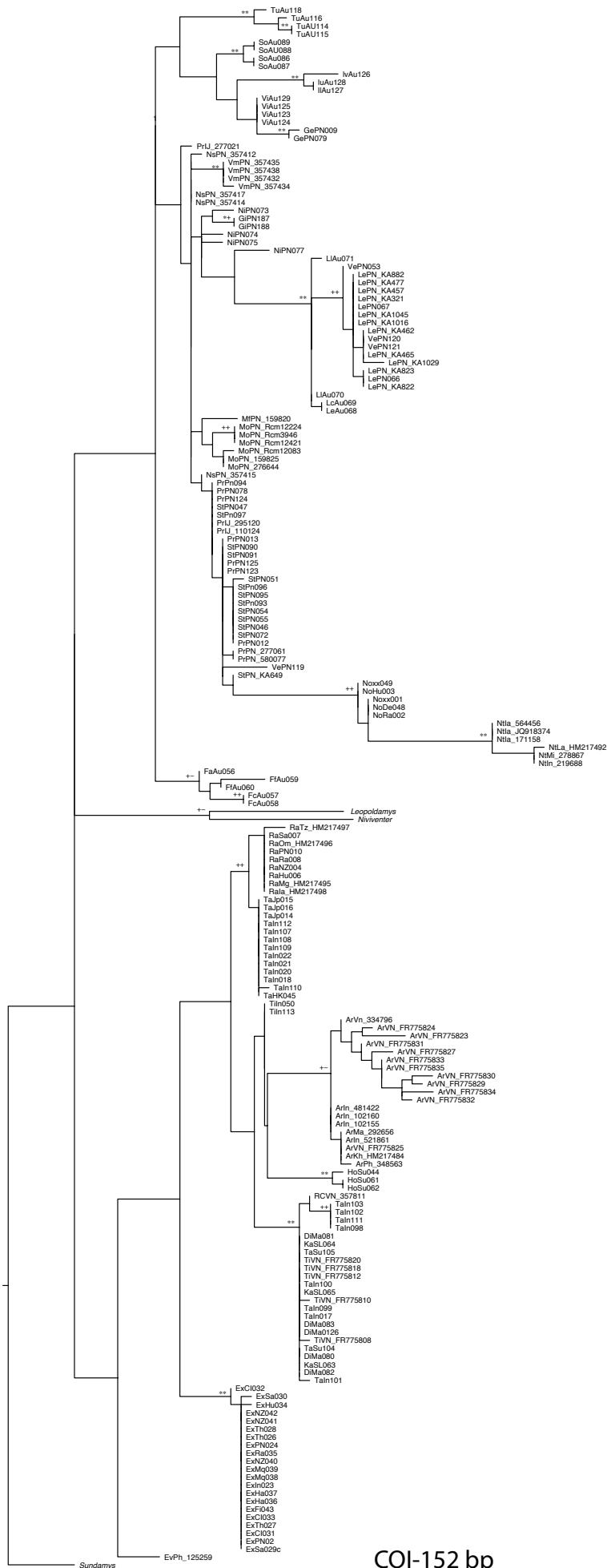

COI-152 bp

0.08

Supplement: Figure S5 — PHYML tree for COI_152. Based on 195 taxa with sequence lengths of 152 bp. Samples are identified. Nodal support is indicated as in Fig. 6. (PDF) [file pone.0098002.s005.pdf]

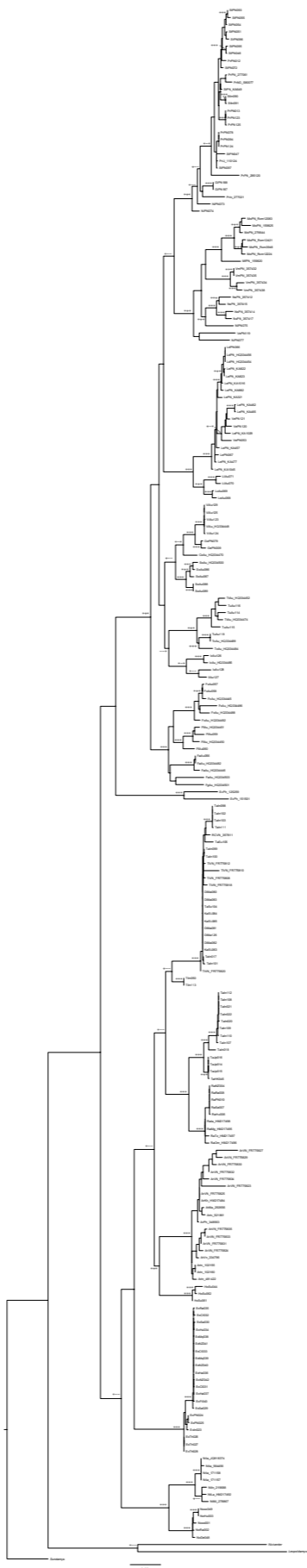

D-loop & COI 1217 bp

Supplement: Figure S6 — RAxML tree for D-loop+COI. The combined dataset of all 217 samples including all the D-loop and COI sequences. Samples are identified and bootstrap support from RAxML is shown. In addition the support for nodes also present in the D-Loop tree (Fig. 3 and Fig. S4) is shown. The levels of support are indicated as follows: * = 90–100% bootstrap or ≥0.95 posterior probability, + = 70–89% bootstrap or 0.80–0.95 posterior probability. The symbol order is RAxML combined tree/RAxML D-loop tree/MrBayes D-loop tree. (PDF) [file pone.0098002.s006.pdf]
