# Supplementary material for: Phylogenetic Species Identification in Rattus Highlights Rapid Radiation and Morphological Similarity of New Guinean Species
Source: PLoS One. 2014 May 27;9(5):e98002. doi: 10.1371/journal.pone.0098002 (PMC4035291; doi:10.1371/journal.pone.0098002)
Supplement: Table S2 — Previously published sequences. (PDF) [file pone.0098002.s008.pdf]

Table S2. Previously published sequences.

Accession denotes the number assigned by the Museum or the collector. The prefix ABTC indicates the Australian Biological Tissue Collection, South Australian Museum,. * indicates that no sequence is available. Ref indicates reference and • denotes an unpublished sequence from GenBank.

Species	Code in analyses	Accession	Location	GenBank Accession		Ref
				D-loop	COI	
R. argentiventer	ArKh_HM217484		Cambodia, Veal Renh	*	HM217484	[1]
R. argentiventer	ArVN_FR775823		Vietnam, Vinh Long	*	FR775823	[2]
R. argentiventer	ArVN_FR775824		Vietnam, Vinh Long	*	FR775824	[2]
R. argentiventer	ArVN_FR775825		Vietnam, Vinh Long	*	FR775825	[2]
R. argentiventer	ArVN_FR775827		Vietnam, Vinh Long	*	FR775827	[2]
R. argentiventer	ArVN_FR775829		Vietnam, Vinh Long	*	FR775829	[2]
R. argentiventer	ArVN_FR775830		Vietnam, Vinh Long	*	FR775830	[2]
R. argentiventer	ArVN_FR775831		Vietnam, Vinh Long	*	FR775831	[2]
R. argentiventer	ArVN_FR775832		Vietnam, Vinh Long	*	FR775832	[2]
R. argentiventer	ArVN_FR775833		Vietnam, Vinh Long	*	FR775833	[2]
R. argentiventer	ArVN_FR775834		Vietnam, Vinh Long	*	FR775834	[2]

Species	Code in analyses	Accession	Location	GenBank Accession		Ref
				D-loop	COI	
R. argentiventer	ArVN_FR775835		Vietnam, Vinh Long	*	FR775835	[2]
R. colletti	Co_HQ334470	ABTC51642	Australia, Northern Territories, Berrimah	HQ334470	*	[3]
R. exulans	ExNZ042		New Zealand, Chatham Islands	EF186311	EF186541	[4]
R. exulans	ExFi043		Fiji	EF186302	EF186532	[4]
R. exulans	ExHa036		Hawaii	EF186303	EF186533	[4]
R. exulans	ExHa037		Hawaii	EF186304	EF186534	[4]
R. exulans	ExHu034		Society Islands, Huahine	EF186305	EF186535	[4]
R. exulans	ExNZ041		New Zealand, Kapiti Island	EF186310	EF186540	[4]
R. exulans	ExRa035		Society Islands, Raiatea	EF186314	EF186544	[4]
R. exulans	ExNZ040		New Zealand, Great Barrier Island	EF186309	EF186539	[4]
R. exulans	ExCI033		Cook Islands, Takutea	EF186301	EF186531	[4]
R. exulans	ExMq038		Marquesas Islands, UaHuka	EF186307	EF186537	[4]

Species	Code in analyses	Accession	Location	GenBank Accession		Ref
				D-loop	COI	
R. exulans	ExMq039		Marquesas Islands, UaHuka	EF186308	EF186538	[4]
R. exulans	ExTh028	ABTC8480	Thailand	EF186319	EF186549	[4]
R. exulans	ExTh027	ABTC8559	Thailand	EF186318	EF186548	[4]
R. exulans	ExPN025	ABTC48895	Papua New Guinea, Madang Province, Nagada Harbour	EF186313	EF186543	[4]
R. exulans	ExCI031		Cook Islands, Aitutaki	EF186299	EF186529	[4]
R. exulans	ExCI032		Cook Islands, Aitutaki	EF186300	EF186530	[4]
R. exulans	ExSa029		Samoa, Manua	EF186315	EF186545	[4]
R. exulans	ExSa030		Samoa, Manua	EF186316	EF186546	[4]
R. exulans	ExTh026	ABTC8553	Thailand	EF186317	EF186547	[4]
R. exulans	ExPN024	ABTC43078	Papua New Guinea, Yuro	EF186312	EF186542	[4]
R. exulans	ExIn023	ABTC48011	Indonesia, Java, Cibodas Forest	EF186306	EF186536	[4]
R. fuscipes assimilis	FaAu056	ABTC51715	Australia, New South Wales, Lismore	EF186321	EF186550	[4]

Species	Code in analyses	Accession	Location	GenBank Accession		Ref
				D-loop	COI	
R. fuscipes assimilis	FcAu_HQ334446		Australia, New South Wales, Mount Nullum Nature Reserve	HQ334446	*	[3]
R. fuscipes assimilis	FcAu_HQ334482		Australia, New South Wales, Rummery Park	HQ334482	*	[3]
R. fuscipes assimilis	FcAu_HQ334503		Australia, Victoria, Wilson's Promontory NP	HQ334503	*	[3]
R. fuscipes coracius	FcAu058	ABTC51735	Australia, Queensland, Atherton Tablelands	EF186323	EF186552	[4]
R. fuscipes coracius	FcAu057	ABTC51727	Australia, Queensland, Mt Windsor	EF186322	EF186551	[4]
R. fuscipes coracius	FcAu_HQ334486		Australia, Queensland, Kahlpalim Track, Davies Creek State Forest	HQ334486	*	[3]
R. fuscipes coracius	FcAu_HQ334488		Australia, Queensland, Kahlpalim Track, Davies Creek State Forest	HQ334488	*	[3]

Species	Code in analyses	Accession	Location	GenBank Accession		Ref
				D-loop	COI	
R. fuscipes coracius	FcAu_HQ334498		Australia, Queensland, Wallaman Falls Rd, Girringun NP	HQ334498	*	[3]
R. fuscipes coracius	FcAu_HQ334445		Australia, Queensland, Mt Windsor - Site A	HQ334445	*	[3]
R. fuscipes fuscipes	FfAu060	ABTC 8605	Australia, Western Australia, Greenhead	EF186325	EF186554	[4]
R. fuscipes fuscipes	FfAu059	ABTC 8615	Australia, Western Australia, Manjimup	EF186324	EF186553	[4]
R. fuscipes fuscipes	FfAu_HQ334450	ABTC8616	Australia, Western Australia, Manjimup	HQ334450	*	[3]
R. fuscipes fuscipes	FfAu_HQ334451	ABTC8644	Australia, Western Australia, Greenhead	HQ334451	*	[3]
R. fuscipes greyi	FgAu_HQ334501		Australia, South Australia, Scott Creek	HQ334501	*	[3]
R. hoffmanni	HoSu062	ABTC65753	Indonesia, Sulawesi, Tangoa	EF186328	EF186558	[4]

Species	Code in analyses	Accession	Location	GenBank Accession		Ref
				D-loop	COI	
R. hoffmanni	HoSu044	ABTC65754	Indonesia, Sulawesi, Tangoa	EF186326	EF186556	[4]
R. hoffmanni	HoSu061	ABTC65809	Indonesia, Sulawesi, Mt Nokilalaki	EF186327	EF186557	[4]
R. kandianus	KaSL064	ABTC8536	Sri Lanka	EF186330	EF186560	[4]
R. kandianus	KaSL065	ABTC8540	Sri Lanka	EF186331	EF186561	[4]
R. kandianus	KaSL063	ABTC8529	Sri Lanka	EF186329	EF186559	[4]
R. leucopus	LePN067	ABTC45966	Papua New Guinea, Northern Province, Ioma	EF186337	EF186565	[4]
R. leucopus	LeAu068	ABTC51766	Australia, Queensland, Millaa Millaa	EF186332	EF186563	[4]
R. leucopus cooktownensis	LcAu069	ABTC8447	Australia, Queensland, Mt Simons	EF186333	EF186562	[4]
R. leucopus dobodurae	LdPN066	ABTC42808	Papua New Guinea, Chimbu Province, Haia	EF186336	EF186564	[4]
R. leucopus dobodurae	LdPN_HQ334454	ABTC42802	Papua New Guinea, Chimbu Province, Haia	HQ334454	*	[3]

Species	Code in analyses	Accession	Location	GenBank Accession		Ref
				D-loop	COI	
R. leucopus dobodurae	LdPN_HQ334456	ABTC42806	Papua New Guinea, Chimbu Province, Haia	HQ334456	*	[3]
R. leucopus leucopus	LlAu070	ABTC8544	Australia, Queensland	EF186334	EF186566	[4]
R. leucopus leucopus	LlAu071	ABTC8493	Australia, Queensland, Rocky River	EF186335	EF186567	[4]
R. lutreolus	luAu128	ABTC27458	Australia, South Australia, Jervois	GU570667	GU570677	[5]
R. lutreolus lutreolus	llAu127	ABTC51720	Australia, New South Wales, Myall Lakes	GU570666	GU570676	[5]
R. lutreolus velutinus	lvAu126	ABTC51762	Australia, Tasmania, Derwent Valley	GU570661	GU570661	[5]
R. lutreolus velutinus	lvAu_HQ334485		Australia, Tasmania, Hobart, Univ. Tasmania	HQ334485	*	[3]
R. niobe	NiPN075	ABTC42503	Papua New Guinea, Central Province, Mt Albert Edward, near Kosipe	EF186344	EF186574	[4]

Species	Code in analyses	Accession	Location	GenBank Accession		Ref
				D-loop	COI	
R. niobe	NiPN074	ABTC43906	Papua New Guinea, Chimbu Province, Mt Karimui	EF186343	EF186573	[4]
R. niobe	NiPN073	ABTC45409	Papua New Guinea, Southern Highlands Province, Magidobo	EF186342	EF186572	[4]
R. niobe	NiPN077	ABTC47145	Papua New Guinea, West Sepik Province, Sol River	EF186345	EF186575	[4]
R. nitidus	NiIa_JQ918374	344	India	*	JQ918374	•
R. nitidus	NiLa_HM217492	L0180	Laos, Luang Prabang	*	HM217492	[1]
R. norvegicus	NoRa002		Society Islands, Raiatea	EF186347	EF186577	[4]
R. norvegicus	NoHu003		Society Islands, Huahine	EF186346	EF186576	[4]
R. norvegicus	NoDe048		Denmark, Copenhagen	AJ428514	AJ428514	[6]
R. norvegicus	Noxx049		laboratory strain	AY172581	AY172581	•
R. norvegicus[*]	Noxx001		laboratory strain	NC_001665	NC_001665	•
R. praetor	PrPN012	ABTC44065	Papua New Guinea, West Sepik Province, Wigote	EF186348	EF186578	[4]

Species	Code in analyses	Accession	Location	GenBank Accession		Ref
				D-loop	COI	
R. praetor	PrPN013	ABTC47232	Papua New Guinea, West Sepik Province, Munbil	EF186349	EF186579	[4]
R. praetor	PrPN125	ABTC47307	Papua New Guinea, West Sepik Province, Munbil	EF186353	EF186583	[4]
R. praetor	PrPN078	ABTC47343	Papua New Guinea, West Sepik Province, Munbil	EF186350	EF186580	[4]
R. praetor	PrPN123	ABTC47271	Papua New Guinea, West Sepik Province, Munbil	EF186351	EF186581	[4]
R. praetor	PrPN124	ABTC47273	Papua New Guinea, West Sepik Province, Munbil	EF186352	EF186582	[4]
R. praetor	PrPN094	ABTC47224	Papua New Guinea, West Sepik Province, Munbil	EF186371	EF186601	[4]
R. rattus	RaHu006		Society Islands, Huahine	EF186354	EF186584	[4]
R. rattus	RaSa007		Samoa	EF186360	EF186590	[4]
R. rattus	RaRa008		Society Islands, Raiatea	EF186359	EF186589	[4]
R. rattus	RaNZ004		New Zealand, Titirangi	EF186355	EF186585	[4]
R. rattus	RaPN010	ABTC50177	Papua New Guinea, Milne	EF186357	EF186587	[4]

Species	Code in analyses	Accession	Location	GenBank Accession		Ref
				D-loop	COI	
			Bay Province, Sideia Is.			
R. rattus diardi	DiMa081	ABTC64908	Malaysia, Kuala Lumpur	EF186295	EF186525	[4]
R. rattus diardi	DiMa082	ABTC64909	Malaysia, Kuala Lumpur	EF186296	EF186526	[4]
R. rattus diardi	DiMa083	ABTC64910	Malaysia, Kuala Lumpur	EF186297	EF186527	[4]
R. rattus diardi	DiMa126	ABTC64906	Malaysia, Kuala Lumpur	EF186298	EF186528	[4]
R. rattus diardi	DiMa080	ABTC64907	Malaysia, Kuala Lumpur	EF186294	EF186524	[4]
R. sordidus	SoAu089	ABTC41168	Australia, Northern Territory, Sir Edward Pellew Is.	EF186365	EF186595	[4]
R. sordidus	SoAu086	ABTC51622	Australia, Queensland, Ingham	EF186362	EF186592	[4]
R. sordidus	SoAu088	ABTC41164	Australia, Northern Territory, Sir Edward Pellew Is.	EF186364	EF186594	[4]
R. sordidus	SoAu087	ABTC51664	Australia, Queensland, Ingham	EF186363	EF186593	[4]
R. sordidus	SoAu_HQ334500		Australia, Queensland,	HQ334500	*	[3]

Species	Code in analyses	Accession	Location	GenBank Accession		Ref
				**D-loop**	**COI**	
			Innisfail			
R. sordidus gestri	GePN009	ABTC44857	Papua New Guinea, National Capital District, Moitaka	EF186356	EF186586	[4]
R. sordidus gestri	GePN079	ABTC44858	Papua New Guinea, National Capital District, Moitaka	EF186358	EF186588	[4]
R. steini	StPN054	ABTC45756	Papua New Guinea, Southern Highlands Province, Bobole	EF186340	EF186570	[4]
R. steini	StPN072	ABTC49306	Papua New Guinea, Madang Province, Bundi	EF186341	EF186571	[4]
R. steini	StPN046	ABTC48962	Papua New Guinea, Morobe Province, Nokopo	EF186338	EF186568	[4]
R. steini	StPN051	ABTC46853	Papua New Guinea, Southern Highlands Province, Waro	EF186339	EF186569	[4]

Species	Code in analyses	Accession	Location	GenBank Accession		Ref
				**D-loop**	**COI**	
R. steini	StPN055	ABTC43216	Papua New Guinea, Chimbu Province, Yuro	EF186361	EF186591	[4]
R. steini	StPN095	ABTC48963	Papua New Guinea, Morobe Province, Nokopo	EF186372	EF186602	[4]
R. steini	StPN093	ABTC43874	Papua New Guinea, Chimbu Province, Doido	EF186370	EF186600	[4]
R. steini	StPN091	ABTC44037	Papua New Guinea, West Sepik Province, Bogalmin	EF186367	EF186597	[4]
R. steini	StPN097	ABTC44058	Papua New Guinea, West Sepik Province, Wigote	EF186374	EF186604	[4]
R. steini	StPN096	ABTC44797	Papua New Guinea, Southern Highlands Province, Waro	EF186373	EF186603	[4]
R. steini	StPN090	ABTC44036	Papua New Guinea, West Sepik Province, Bogalmin	EF186366	EF186596	[4]
R. steini	StPN047	ABTC49035	Papua New Guinea, Morobe Province, Nokopo	EF186368	EF186598	[4]

Species	Code in analyses	Accession	Location	GenBank Accession		Ref
				D-loop	COI	
R. tanezumi	TaJp015	ABTC 8514	Japan, Amami Island	EF186394	EF186624	[4]
R. tanezumi	TaJp016	ABTC 8562	Japan, Amami Island	EF186395	EF186625	[4]
R. tanezumi	TaIn020	ABTC47981	Indonesia, Java, Yogyakarta	EF186378	EF186608	[4]
R. tanezumi	TaIn021	ABTC47982	Indonesia, Java, Yogyakarta	EF186379	EF186609	[4]
R. tanezumi	TaIn108	ABTC47985	Indonesia, Java, Yogyakarta	EF186388	EF186618	[4]
R. tanezumi	TaIn109	ABTC47986	Indonesia, Java, Yogyakarta	EF186389	EF186619	[4]
R. tanezumi	TaIn110	ABTC47987	Indonesia, Java, Yogyakarta	EF186390	EF186620	[4]
R. tanezumi	TaIn111	ABTC47988	Indonesia, Java, Yogyakarta	EF186391	EF186621	[4]
R. tanezumi	TaIn112	ABTC47989	Indonesia, Java, Yogyakarta	EF186392	EF186622	[4]
R. tanezumi	TaIn017	ABTC47992	Indonesia, Java, Jakarta	EF186375	EF186605	[4]

Species	Code in analyses	Accession	Location	GenBank Accession		Ref
				**D-loop**	**COI**	
R. tanezumi	TaIn098	ABTC47995	Indonesia, Java, Jakarta	EF186381	EF186611	[4]
R. tanezumi	TaIn099	ABTC47996	Indonesia, Java, Jakarta	EF186382	EF186612	[4]
R. tanezumi	TaIn101	ABTC47998	Indonesia, Java, Jakarta	EF186384	EF186614	[4]
R. tanezumi	TaIn102	ABTC47999	Indonesia, Java, Jakarta	EF186385	EF186615	[4]
R. tanezumi	TaSu104	ABTC48004	Indonesia, northern Sulawesi	EF186396	EF186626	[4]
R. tanezumi	FlHk045	ABTC 8489	China, Hong Kong	EF186320	EF186555	[4]
R. tanezumi	TaJp014	ABTC 8487	Amami Island, Japan	EF186393	EF186623	[4]
R. tanezumi	TaIn022	ABTC47983	Indonesia, Java, Yogyakarta	EF186380	EF186610	[4]
R. tanezumi	TaIn107	ABTC47984	Indonesia, Java, Yogyakarta	EF186387	EF186617	[4]
R. tanezumi	TaIn018	ABTC47993	Indonesia, Java, Jakarta	EF186376	EF186606	[4]
R. tanezumi	TaIn100	ABTC47997	Indonesia, Java, Jakarta	EF186383	EF186613	[4]
R. tanezumi	TaIn103	ABTC48000	Indonesia, Java, Jakarta	EF186386	EF186616	[4]
R. tanezumi	TaSu105	ABTC48005	Indonesia, northern Sulawesi	EF186397	EF186627	[4]

Species	Code in analyses	Accession	Location	GenBank Accession		Ref
				D-loop	COI	
R. "*tiomanicus*"	TVN_FR775808		Vietnam, Dong Nai,	*	FR775808	[2]
R. "*tiomanicus*"	TVN_FR775810		Vietnam, Dong Nai,	*	FR775810	[2]
R. "*tiomanicus*"	TVN_FR775812		Vietnam, Vinh Long	*	FR775812	[2]
R. "*tiomanicus*"	TVN_FR775818		Vietnam, Vinh Long	*	FR775818	[2]
R. "*tiomanicus*"	TVN_FR775820		Vietnam, Vinh Long	*	FR775820	[2]
R. tiomanicus	TiIn113	ABTC48025	Indonesia, Java, Cibodas forest	EF186399	EF186629	[4]
R. tiomanicus	TiIn050	ABTC48026	Indonesia, Java, Cibodas forest	EF186398	EF186628	[4]
R. tunneyi	TuAu116	ABTC 8719	Australia, Northern Territory, Nourlangie Camp Road	EF186402	EF186632	[4]
R. tunneyi	TuAu115	ABTC 8737	Australia, Western Australia, Mitchell Plateau	EF186401	EF186631	[4]
R. tunneyi	TuAu114	ABTC29636	Australia, Northern Territory, Litchfield National Park	EF186400	EF186630	[4]

**Species**	**Code in analyses**	**Accession**	**Location**	**GenBank Accession**		**Ref**
				**D-loop**	**COI**	
R. tunneyi	TuAu118	ABTC51688	Australia, Queensland, Yarraman	EF186403	EF186633	[4]
R. tunneyi culmorum	TcAu_HQ334484		Australia, New South Wales, Bundjalong National Park	HQ334484	*	[3]
R. tunneyi culmorum	TcAu_HQ334499		Australia, Queensland, Yarraman	HQ334499	*	[3]
R. tunneyi tunneyi	TtAu_HQ334452	ABTC08815	Australia, Western Australia, Mitchell Plateau	HQ334452	*	[3]
R. tunneyi tunneyi	TtAu_HQ334474	ABTC51684	Australia, Northern Territory, Sir Edward Pellew Is.	HQ334474	*	[3]
R. verecundus	VePN121	ABTC44796	Papua New Guinea, Southern Highlands Province, Waro	EF186407	EF186637	[4]
R. verecundus	VePN053	ABTC45161	Papua New Guinea, Southern Highlands	EF186404	EF186634	[4]

Species	Code in analyses	Accession	Location	GenBank Accession		Ref
				D-loop	COI	
			Province, Bobole			
R. verecundus	VePN119	ABTC49292	Papua New Guinea, Madang Province, Bundi	EF186405	EF186635	[4]
R. verecundus	VePN120	ABTC43510	Papua New Guinea, Chimbu Province, Noru	EF186406	EF186636	[4]
R. villosissimus	ViAu129	ABTC00549	Australia, South Australia	GU570663	GU570663	[4]
R. villosissimus	ViAu123	ABTC 00548	Australia, South Australia, Purni Bore	GU570668	GU570678	[4]
R. villosissimus	ViAu124	ABTC 23632	Australia, south west Queensland, Palparara	GU570669	GU570679	[4]
R. villosissimus	ViAu125	ABTC 41137	Australia, Northern Territory, Sir Edward Pellew Is.	GU570670	GU570680	[4]
R. villosissimus	ViAu_HQ334448	ABTC08439	Australia, Queensland, Midway Tank	HQ334448	*	[3]
Leopoldamys sabanus	Leopoldamys		Thailand, Kanchanaburi	*	HM217531	[1]
Niviventer fulvescens	Niviventer		Thailand, Loei	*	HM217589	[1]

References

1. Pagès M, Chaval Y, Herbreteau V, Waengsothorn S, Cosson J-Fo, et al. (2010) Revisiting the taxonomy of the Rattini tribe: a phylogeny-based delimitation of species boundaries. BMC Evol Biol 10: 184.
2. Balakirev AE, Rozhnov VV (2012) Contribution to the species composition and taxonomic status of some *Rattus* inhabiting Southern Vietnam and Sundaland. Russian Journal of Theriology 11: 33-45.
3. Rowe KC, Aplin KP, Baverstock PR, Moritz C (2011) Recent and rapid speciation with limited morphological disparity in the genus *Rattus*. Syst Biol 60: 188-203.
4. Robins JH, Hingston M, Matisoo-Smith E, Ross HA (2007) Identifying *Rattus* species using mitochondrial DNA. Mol Ecol Notes 7: 717-729.
5. Robins JH, McLenachan PA, Phillips MJ, McComish BJ, Matisoo-Smith E, et al. (2010) Evolutionary relationships and divergence times among the native rats of Australia. BMC Evol Biol 10: 375.
6. Nilsson MA, Gullberg A, Spotorno AE, Arnason U, Janke A (2003) Radiation of extant marsupials after the K/T boundary: evidence from complete mitochondrial genomes J Mol Evol 57: S3-S12.
