# Supplementary material for: Correction: Phylogenetic Species Identification in Rattus Highlights Rapid Radiation and Morphological Similarity of New Guinean Species
Source: PLoS One. 2014 Sep 2;9(9):e107667. doi: 10.1371/journal.pone.0107667 (PMC4152336; doi:10.1371/journal.pone.0107667)
Supplement: Table S1 — Sample Information including GenBank accession numbers for newly published sequences used in this study. (PDF) [file pone.0107667.s001.pdf]

Table S1. Sample Information including GenBank accession numbers for newly published sequences used in this study.

Species designation is that of the Museum or collector. Accession numbers prefixed with AMNH are from the American Museum of Natural History, ABTC the Australian Biological Tissue Collection, South Australian Museum, CM the Australian National Wildlife Collection and USNM the Smithsonian Institution National Museum of Natural History. Those with KA in the analysis code were collected by Ken Aplin. In the D-loop and COI columns the numbers prefixed with KF are GenBank accession numbers; • indicates no PCR attempted; ✗ indicates amplification failure; ✓ indicates successful amplification of a 152 bp fragment of COI but these sequences are too short for inclusion in GenBank and are available in supporting information as fasta files.

**Species**	**Code in analyses**	**Accession**	**Location**	**D-loop**	**COI**		**Tissue**
					**655 bp**	**152 bp**	
R. argentiventer	ArIn_102155	AMNH102155	Indonesia, West Bali, Soember Klampok	KF494834	•	✓	bone
R. argentiventer	ArI_102160	AMNH102160	Indonesia, West Bali, Goengnoeng, Linker, 300m	KF494835	•	✓	bone
R. argentiventer	ArVn_334796	USNM334796	Vietnam	KF494837	•	✓	dried skin
R. argentiventer	ArIn_481422	USNM481422	Indonesia	KF494838	•	✓	dried skin
R. argentiventer	ArIn_521861	USNM521861	Indonesia	KF494839	•	✓	dried skin
R. argentiventer	ArMa_292656	USNM292656	Malaysia	KF494836	•	✓	dried skin
R. argentiventer	ArPh_348563	USNM348563	Philippines	KF494840	•	✓	dried skin

Species	Code in analyses	Accession	Location	D-loop	COI		Tissue
					655 bp	152 bp	
R. argentiventer	Rarg_241083	USNM241083	Thailand	✗	✗	✗	dried skin
R. argentiventer	Rarg_348533	USNM348533	Philippines	✗	✗	✗	dried skin
R. everetti albigularis	EvPh_125259	USNM125259	Philippines, Mindanao	KF494841	•	✓	dried skin
R. everetti everetti	EvPh_151501	USNM151501	Philippines, Luzon	KF494842	•	✗	dried skin
R. giluwensis	GiPN187	ABTC87301	Papua New Guinea, Enga Province, Waile Creek near Porgera, 3100m	KF510069	KF510035	•	tissue in ethanol
R. giluwensis	GiPN188	ABTC87305	Papua New Guinea, Enga Province, Waile Creek near Porgera, 3100m	KF510070	KF510036	•	tissue in ethanol
R. leucopus	LePN_KA321	ABTC130364	Papua New Guinea, Gulf Province, Purari River, vicinity of Wabo	KF510071	KF510037	•	tissue in ethanol
R. leucopus	LePN_KA457	ABTC130439	Papua New Guinea, Gulf Province, Purari River, vicinity of Wabo	KF510072	KF510038	•	tissue in ethanol
R. leucopus	LePN_KA462	ABTC130443	Papua New Guinea, Gulf	KF510073	KF510039	•	tissue in

Species	Code in analyses	Accession	Location	D-loop	COI		Tissue
					655 bp	152 bp	
			Province, Purari River, vicinity of Wabo				ethanol
R. leucopus	LePN_KA465	ABTC130445	Papua New Guinea, Gulf Province, Purari River, vicinity of Wabo	KF510074	KF510040	•	tissue in ethanol
R. leucopus	LePN_KA477	ABTC130453	Papua New Guinea, Gulf Province, Purari River, vicinity of Wabo	KF510075	KF510041	•	tissue in ethanol
R. leucopus	LePN_KA821	ABTC130496	Papua New Guinea, Gulf Province, Purari River, vicinity of Wabo	KF510076	KF510042	•	tissue in ethanol
R. leucopus	LePN_KA823	ABTC130498	Papua New Guinea, Gulf Province, Purari River, vicinity of Wabo	KF510077	KF510043	•	tissue in ethanol
R. leucopus	LePN_KA882	ABTC130519	Papua New Guinea, Gulf Province, Purari River, vicinity of Wabo	KF510078	KF510044	•	tissue in ethanol

Species	Code in analyses	Accession	Location	D-loop	COI		Tissue
					655 bp	152 bp	
R. leucopus	LePN_KA1016	ABTC130585	Papua New Guinea, Gulf Province, Purari River, vicinity of Wabo	KF510079	KF510045	•	tissue in ethanol
R. leucopus	LePN_KA1029	ABTC130594	Papua New Guinea, Gulf Province, Purari River, vicinity of Wabo	KF510080	KF510046	•	tissue in ethanol
R. leucopus	LePN_KA1045	ABTC130600	Papua New Guinea, Gulf Province, Purari River, vicinity of Wabo	KF510081	KF510047	•	tissue in ethanol
R. mordax	MoPN_Rcm3946	CM3946	Papua New Guinea, Central Province, Efogi	KF494843	•	✓	bone
R. mordax	MoPN_Rcm12083	CM12083	Papua New Guinea, Milne Bay Province, Nowata	KF494844	•	✓	bone
R. mordax	MoPN_Rcm12224	CM12224	Papua New Guinea, Central Province, Nunumai	KF494845	•	✓	bone
R. mordax	MoPN_Rcm12421	CM12421	Papua New Guinea, Central Province, Mori River	KF494846	•	✓	bone

Species	Code in analyses	Accession	Location	D-loop	COI 655 bp	COI 152 bp	Tissue
R. mordax	MoPN_159825	AMNH159825	Papua New Guinea, Milne Bay Province, Woodlark Island, Kulumadau	KF494847	•	✓	bone
R. mordax	MoPN_276644	USNM276644	Papua New Guinea, Milne Bay Province, Milne Bay	KF494848	•	✓	dried skin
R. mordax fergussoniensis	MfPN_159820	AMNH159820	Papua New Guinea, Milne Bay Province, Fergusson Island, Iamelele	KF494849	•	✓	bone
R. niobe	NsPN_357412	USNM357412	Papua New Guinea, Morobe Province, Wau, 1600-2300m	KF510050	•	✓	dried skin
R. niobe	NsPN_357414	USNM357414	Papua New Guinea, Morobe Province, Wau, 1600-2300m	KF510051	•	✓	dried skin
R. niobe	NsPN_357415	USNM357415	Papua New Guinea, Morobe Province, Wau, 1600-2300m	KF510052	•	✓	dried skin
R. niobe	NsPN_357417	USNM357417	Papua New Guinea, Morobe Province, Wau, 1600-2300m	KF510053	•	✓	dried skin
R. nitidus	NtIa_171157	AMNH171157	India, Assam State, Khasia	KF510054	✗	✗	bone

Species	Code in analyses	Accession	Location	D-loop	COI		Tissue
					655 bp	152 bp	
			Hills, Nongpoh				
R. nitidus	NtIa_171158	AMNH171158	India, Assam State, Khasia Hills, Nongpoh	KF510055	•	✓	bone
R. nitidus	NtIn_219688	USNM219688	Indonesia	KF510056	•	✓	dried skin
R. nitidus	NtMi_278867	USNM278867	Micronesia, Belau also known as Palau	KF510057	•	✓	dried skin
R. nitidus	NtIa_564456	USNM564456	India	KF510059	•	✓	dried skin
R. nitidus	NtPh_145803	USNM145803	Philippines	✗	✗	✗	dried skin
R. nitidus	NtCn_252912	USNM252912	China	✗	✗	✗	dried skin
R. praetor	PrIJ_110124	AMNH110124	Indonesia, Papua Province, Hollandia	KF510060	•	✓	bone
R. praetor	PrIJ_277021	USNM277021	Indonesia, Papua Province, Bird's Head, Sansapor	KF510061	•	✓	dried skin
R. praetor	PrIJ_295120	USNM295120	Indonesia, Jayapura, Papua Province	KF510062	•	✓	dried skin
R. praetor praetor	PrPN_277061	USNM277061	Papua New Guinea, Bougainville Island	KF510063	•	✓	dried skin

Species	Code in analyses	Accession	Location	D-loop	COI		Tissue
					**655 bp**	**152 bp**	
R. praetor praetor	PrPN_580077	USNM580077	Papua New Guinea (New Ireland)	KF510064	•	✓	dried skin
R. praetor	Rprae_277053	USNM277053	Papua New Guinea Bougainville Island	✗	✗	✗	dried skin
R. ratus Complex	RCVN_357811	USNM357811	Vietnam	KF510058	•	✓	dried skin
R. steini	StPN_KA649	ABTC128624	Papua New Guinea, Morobe Province, Nambonga River	KF510082	KF510048	•	tissue in ethanol
R. verecundus mollis	VmPN_357432	USNM357432	Papua New Guinea, Morobe Province, Wau, 1700-1950m	KF510065	•	✓	dried skin
R. verecundus mollis	VmPN_357434	USNM357434	Papua New Guinea, Morobe Province, Wau, 1700-1950m	KF510066	•	✓	dried skin
R. verecundus mollis	VmPN_357435	USNM357435	Papua New Guinea, Morobe Province, Wau, 1700-1950m	KF510067	•	✓	dried skin
R. verecundus mollis	VmPN_357438	USNM357438	Papua New Guinea, Morobe Province, Wau, 1700-1950m	KF510068	•	✓	dried skin
Sundamys muelleri	Sundamys	ABTC117777	Indonesia	KF510083	KF510049	•	tissue in ethanol
